# Global burden and projections of chronic kidney disease attributable to chronic glomerulonephritis in women of childbearing age

**DOI:** 10.1097/MD.0000000000049583

**Published:** 2026-07-17

**Authors:** Run-Ze Wang, Ye-Xin Chen, Yi-Shan Wu, Bei-Bei Ye, Mao-Xuan Lin, Dong-Sen Hu, Zhao-Xi Dong, Zhe-Yu Xu, Jia-You Liu, Ji-Yuan Hu, Hong-Fang Liu

**Affiliations:** a Nephrology and Endocrinology Department, Dongzhimen Hospital, Beijing University of Chinese Medicine, Beijing, China; b Nephrology and Endocrinology Department, Beijing University of Chinese Medicine, Beijing, China; c Endocrinology Department, Guang’anmen Hospital of China Academy of Chinese Medical Sciences, Beijing, China; d Nephrology and Endocrinology Department, Beijing Hospital of Integrated Traditional Chinese and Western Medicine, Beijing, China.

**Keywords:** chronic kidney disease, epidemiology, Global Burden of Disease, women of childbearing age

## Abstract

Glomerulonephritis (GN) is a leading cause of chronic kidney disease (CKD) and disproportionately affects women of childbearing age (WCBA). Despite this vulnerability, global data on CKD attributable to GN in this population remain scarce. This study analyzes the disease burden from 1990 to 2021 and projects trends through 2049 across age groups, regions, and countries. Data were sourced from the Global Burden of Disease Study 2021 (GBD 2021), focusing on incidence and disability-adjusted life years for CKD due to GN in WCBA. Age-standardized rates (ASIR/ASDR) were calculated. Trends were assessed using Joinpoint regression, and the Bayesian age-period-cohort model forecast future burden. Regions were categorized by Socio-Demographic Index (SDI) levels. Health inequalities were evaluated using slope and concentration indices, with frontier analysis identifying priority countries. From 1990 to 2021, the global burden of CKD attributable to GN in WCBA increased. ASIR and disability-adjusted life years peaked in the 45 to 49 age group, while the highest incidence occurred in the 15 to 19 group. Health inequalities persist; high and high-middle SDI regions bear a lower burden compared to middle and low-middle SDI regions. Geographically, Western Europe and high-income Central Asia had the lightest burden, while Central America and the Asia-Pacific region faced the heaviest. Notably, the global burden has accelerated over the past 3 years, and projections indicate a continued rise through 2049. This study highlights the increasing burden of CKD due to GN among WCBA, particularly in middle and low SDI regions. Strengthened global collaboration and enhanced access to diagnostic and therapeutic technologies in low-SDI areas are urgently needed. Greater attention must also be paid to younger and older WCBA subgroups experiencing higher burdens. Given the rapid recent growth, vulnerable groups such as WCBA require increased focus in kidney disease research and policy.

## 1. Introduction

Glomerulonephritis (GN) is a common glomerular disease that predominantly affects middle-aged and young individuals. It represents a significant public health concern and is one of the leading contributors to the development of chronic kidney disease (CKD). Approximately 20% of CKD cases are attributable to GN, placing a substantial burden on the socioeconomic landscape.^[1]^

Women of childbearing age (WCBA), defined as females aged 15 to 49 years, encompass a stage of development characterized by reproductive capability and cyclical hormonal changes, including pregnancy. During this period, women should prioritize issues related to breast health, cervical health, and contraception.^[2]^ However, misconceptions regarding reproductive health are common in this population, and maternal mental health during pregnancy warrants particular attention.^[3]^ Additionally, women in this group often face unique social and economic pressures, such as work-related burdens and family responsibilities, which may further affect their overall well-being.

CKD is especially relevant to WCBA, with recent studies indicating that nearly 4% of women in this demographic are affected by the condition.^[4]^ CKD also significantly impacts pregnancy outcomes in WCBA, as it is frequently associated with complications such as sexual dysfunction, reduced fertility, abnormal uterine bleeding, and high-risk pregnancies.^[5]^ During pregnancy, physiological changes occur in the kidneys, including an increase in glomerular filtration rate, enhanced urinary protein excretion, and alterations in tubular function.^[6]^ Moreover, WCBA with underlying CKD face an increased risk of preterm birth and preeclampsia, with their fetuses being more vulnerable to fetal growth restriction.^[7,8]^ As a major cause of CKD, GN should be of particular concern in this context. Women with GN may be at an increased risk of adverse pregnancy outcomes, including preterm birth, preeclampsia, and low birth weight, while pregnancy itself may influence the progression of GN. A cohort study on renal pathology in pregnant women demonstrated that extensive glomerular disease is frequently detected through kidney biopsy during pregnancy or within one year postpartum.^[9]^ Furthermore, the rate of glomerular filtration rate decline during follow-up was faster in the affected group compared to controls, suggesting that WCBA are more susceptible to the development of CKD, with more severe glomerular damage.

Previous Global Burden of Disease (GBD) analyses have characterized the global burden of chronic kidney disease (CKD) and its major etiologies within the general population.^[10,11]^ Concurrently, a recent study based on GBD 2021 has further explored the disease burden and projections of CKD attributable to GN across all age populations.^[12]^ However, there is currently a lack of global overview and analysis regarding the disease burden of CKD attributable to GN in WCBA, underscoring the insufficient attention given to this specific population. This study systematically analyzes the distribution characteristics, temporal trends, and future projections of the burden of CKD attributable to chronic GN in WCBA, while exploring the underlying reasons for disparities in disease burden. It proposes plausible hypotheses: persistent health inequities, along with factors such as age structure, SDI levels, and healthcare policies, continue to influence the disease burden, and the burden is expected to rise further in the future. This analysis provides important epidemiological evidence for developing effective prevention and management strategies for this specific population.

## 2. Materials and methods

### 2.1. Data sources

To investigate the disease burden of CKD attributable to GN in WCBA, this study utilized data from the Global Burden of Disease Study 2021 (GBD 2021; https://ghdx.healthdata.org/gbd-2021/). The GBD 2021, compiled by the Institute for Health Metrics and Evaluation, offers a comprehensive epidemiological analysis of the burden of 371 diseases and injuries across 204 countries and regions, with all data freely accessible and downloadable. As a comprehensive macro-level research initiative, the GBD database employs standardized protocols to systematically recompile and recalibrate all raw data from diverse sources, ensuring comparability across datasets. This process ultimately generates statistical estimates accompanied by uncertainty intervals (UI).

For this analysis, we focused specifically on CKD attributable to GN. Regarding age groups, the study examined data for the “15–49 years” and “age-standardized” categories, as well as the following specific age groups: “15–19 years” “20–24 years” “25–29 years” “30–34 years” “35–39 years” “40–44 years” and “45–49 years.” As for the burden indicators, we selected incidence and disability-adjusted life years (DALYs) for evaluation. Additionally, the study explored the relationship between the level of economic development and CKD attributable to GN in WCBA.

### 2.2. Statistical analysis

This study incorporated two key metrics: quantity and rate. The quantity represents the raw case numbers from the GBD 2021, while the rate refers to the number of cases per 1,00,000 population. All raw values reported by GBD 2021 are accompanied by 95% UI, which were derived through the aggregation and weighting process used in the GBD 2021 study.

The study employed age-standardized rates^[13]^ for data analysis. Given that the data are stratified by specific age groups, age-standardized rates were calculated using direct standardization. This process involves multiplying the crude rate for each age group (typically in 5-year intervals) by the proportion of the population in that group within the standard population, followed by summing the results. DALYs and incidence rates were selected as the outcome measures to provide a comprehensive assessment of the disease burden. To explore the relationship between CKD attributable to GN in WCBA and socioeconomic development, the study integrated the Socio-Demographic Index (SDI) data from the GBD 2021, spanning from 1990 to 2021. The SDI values were categorized into 5 groups based on varying levels of economic development: low (<0.46), low-middle (0.46–0.60), middle (0.61–0.69), high-middle (0.70–0.81), and high (>0.81).

The Joinpoint regression program in R software (version 4.4.1; R Foundation for Statistical Computing) was employed to assess trends in disease burden from 1990 to 2021, calculating the annual percentage change (APC) and the average annual percentage change (AAPC).^[14,15]^ The correlation between the SDI and the disease burden of CKD attributable to GN in WCBA was calculated using R’s correlation coefficient function. The slope index and concentration index, which represent absolute and relative health inequalities, were derived using the “inequality” package. This study conducted a frontier analysis to explore the disease burden control of CKD attributable to GN in WCBA across different countries based on their SDI levels.^[10]^ The boundary line represents the countries and regions with the lowest disease burden at their respective SDI levels. The farther a country is from the boundary line, the greater the disparity between its actual disease burden and the potential achievable disease burden. The results of the frontier analysis identified the 5 countries closest to the frontier line in lower SDI regions, which were highlighted in blue, and the 5 countries closest to the frontier line in higher SDI regions, marked in red. These countries exhibit policies and treatment approaches for disease management at their SDI level that could serve as valuable references. Among all countries, the 15 countries furthest from the frontier line were denoted in black, indicating that these countries need to strengthen the management of CKD attributable to GN in WCBA in order to reduce the disease burden. The Bayesian age-period-cohort (BAPC) and Integrated Nested Laplace Approximation packages in R were utilized to analyze the incidence and DALYs of CKD attributable to GN in WCBA across different regions and age groups from 2022 to 2049, with age-standardized incidence rates (ASIR) and age-standardized death rates (ASDR) being computed. Compared to other predictive models, the BAPC model is capable of integrating data from various age groups for comprehensive analysis and demonstrates a lower error rate, enabling more accurate and comprehensive predictions of disease burden trends.^[16]^

All statistical analyses and data visualizations were performed using Stata 16.0 (StataCorp LLC, College Station) and R 4.4.1 (R Foundation for Statistical Computing, Vienna, Austria). UIs were defined as the 2.5th and 97.5th percentiles of the posterior distribution, with differences exceeding 95% considered statistically significant.^[17]^ A *P*-value of<.05 was regarded as statistically significant.

## 3. Result

### 3.1. The disease burden of CKD attributable to GN in WCBA across different age groups

In 2021, the global incidence and DALYs of CKD attributable to GN in WCBA were 32,409 (95% UI, 37,743–26,705) and 12,11,463 (95% UI, 14,42,748–10,08,547), respectively. Among different age groups, the highest case and ASDR occurred in the 45 to 49 years age group (case: 2,10,641, 95% UI 2,87,154–1,45,327; ASDR: 89.39 per 1,00,000 population, 95% UI 121.86–61.67). Conversely, the lowest ASDR was found in the 15 to 19 years age group, at 41.46 per 1,00,000 population (95% UI, 53.92–29.72). The highest number of cases occurred in the 15 to 19 years age group (5615, 95% UI 8480–3150). The highest ASIR was observed in the 45 to 49 years age group, at 2.11 per 1,00,000 population (95% UI, 2.69–1.46). This indicated a significant disease burden in this group, warranting focused attention. The lowest number of cases was found in the 25 to 29 years age group, with only 3595 (95% UI, 5900–1837), and the lowest ASIR was also observed in this group, at 1.24 per 1,00,000 population (95% UI, 2.03–0.63; Table 1).

**Table 1:** Cases, ASR, and AAPC of CKD attributable to GN in WCBA by global, SDI regions, and age groups in 2021.

	Incidents			DALYs		
	Cases (95% UI)	ASR (95% UI)	AAPC (95% UI)	Cases (95% UI)	ASR (95% UI)	AAPC (95% UI)
Global	32,409 (26,705, 5,37,743)	1.66 (1.37, 1.94)	0.7 (0.67, 0.72)	12,11,463(10,08,547, 14,42,748)	62.16 (51.75, 74.03)	0.46 (0.25, 0.68)
SDI regions						
High SDI	2869 (1678, 4325)	1.1 (0.62, 1.7)	0.6 (0.52, 0.68)	86,141 (67,451, 1,08,496)	32.36 (25.35, 40.77)	1.23 (1.05, 1.42)
High-middle SDI	4478 (2631, 6697)	1.42 (0.81, 2.17)	0.71 (0.64, 0.77)	73,911 (53,349, 98,258)	22.68 (16.35, 30.26)	−1.13 (−1.32, −0.94)
Low SDI	3965 (2262, 6061)	1.45 (0.85, 2.18)	0.39 (0.34, 0.44)	2,89,274 (2,02,796, 3,92,559)	110.36 (77.04, 150.29)	−0.29 (−0.47, −0.11)
Low-middle SDI	9300 (5510, 13,768)	1.85 (1.1, 2.72)	0.48 (0.39, 0.57)	3,75,911 (2,65,589, 5,13,123)	76 (53.62, 103.81)	0.43 (0.27, 0.59)
Middle SDI	11,767 (7309, 16,836)	1.89 (1.16, 2.73)	0.8 (0.77, 0.82)	3,84,904 (2,74,930, 5,13,920)	60.44 (43.29, 80.64)	0.16 (−0.02, 0.35)
Ages						
15–19 yr	5615 (3150, 8485)	1.85 (1.04, 2.79)	0.72 (0.7, 0.74)	1,25,882 (90,244, 1,63,740)	41.46 (29.72, 53.92)	0.08 (0.05, 0.11)
20–24 yr	4208 (2290, 6325)	1.43 (0.78, 2.15)	0.57 (0.5, 0.65)	1,56,938 (1,15,376, 2,09,272)	53.43 (39.28, 71.24)	0.41 (0.26, 0.55)
25–29 yr	3595 (1837, 5900)	1.24 (0.63, 2.03)	0.47 (0.44, 0.49)	1,60,822 (1,22,118, 2,08,655)	55.27 (41.97, 71.71)	0.3 (0.19, 0.42)
30–34 yr	4087 (2454, 6003)	1.37 (0.82, 2.01)	0.56 (0.54, 0.58)	1,80,543 (1,32,715, 2,34,388)	60.4 (44.4, 78.41)	0.17 (−0.05, 0.38)
35–39 yr	4907 (3089, 6636)	1.77 (1.11, 2.39)	0.78 (0.74, 0.82)	1,80,871 (1,33,436, 2,37,903)	65.11 (48.03, 85.64)	0.34 (0.12, 0.56)
40–44 yr	5032 (3593, 6715)	2.03 (1.45, 2.71)	0.73 (0.7, 0.77)	1,95,766 (1,42,061, 2,55,692)	78.91 (57.26, 103.06)	0.48 (0.27, 0.7)
45–49 yr	4965 (3435, 6330)	2.11 (1.46, 2.69)	0.59 (0.55, 0.63)	2,10,641 (1,45,327, 2,87,154)	89.39 (61.67, 121.86)	0.49 (0.33, 0.65)

AAPC = average annual percentage change, ASDR = age-standardized disability-adjusted life years rate, ASIR = age-standardized incidence rate, ASR = age-standardized rate, CKD = chronic kidney disease, DALY = disability-adjusted life year, GN = glomerulonephritis, SDI = Socio-demographic Index, WCBA = women of childbearing age.

In 2021, the global incident cases of CKD attributable to GN in WCBA were 32,409 (95% UI, 26,705–37,743), and the total DALYs were 12,11,463 (95% UI, 10,08,547–14,42,748). The incidence peaked in the 15 to 19 years age group (5615 cases, 95% UI 3150–8480), whereas the highest ASIR was observed in the 45 to 49 years age group (2.11 per 1,00,000 population, 95% UI 1.46–2.69). DALY burden increased with age, with the highest DALY counts and age‑standardized DALY rate (ASDR) in the 45 to 49 years age group (DALYs: 2,10,641, 95% UI 1,45,327–2,87,154; ASDR: 89.39 per 1,00,000 population, 95% UI 61.67–121.86; Table 1).

**Figure 1.**
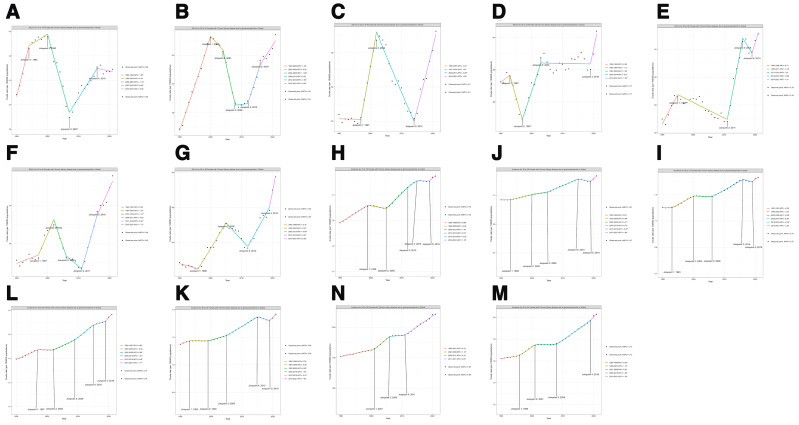
AAPC of CKD attributable to GN in WCBA for different ages from 1990 to 2021 based on join point regression model (Table S1, Supplemental Digital Content 1). (A–G) ASDR of CKD attributable to GN in WCBA, (H–N) ASIR of CKD attributable to GN in WCBA. AAPC = average annual percentage change, ASDR = age-standardized DALYs rate, ASIR = age-standardized incidence rate, CKD = chronic kidney disease, DALY = disability-adjusted life year, GN = glomerulonephritis, SDI = Socio-demographic Index, WCBA = women of childbearing age.

The trend of CKD attributable to GN in WCBA from 1990 to 2021 was assessed using a Joinpoint regression model, and the APC and AAPC were calculated (Fig. 1A–N, Table S1, Supplemental Digital Content 1). Both ASIR and ASDR exhibited an increasing trend across all age groups over 32 years. The ASIR for all age groups steadily increased over the past 30 years, with nearly identical growth trends across age groups. However, DALYs fluctuated over the 30-year period, with noticeable increases and decreases across different years, and trends varied significantly across age groups. The most rapid increase in ASDR occurred in the 45 to 49 years age group, with a rate of 0.49 per 1,00,000 population (95% UI, 0.33–0.65). In terms of APC, from 2019 to 2021, the APC of ASIR across all age groups was the highest in the past 31 years. With the exception of the 15 to 19 years age group, where the APC of DALYs showed a slight decline (−0.06), the AAPC of DALYs in other age groups remained relatively high. This suggests that, without further interventions, the disease burden of CKD attributable to GN in WCBA is likely to increase substantially in the future. These findings suggest that the disease burden in these age groups warrants particular attention.

### 3.2. The disease burden of CKD attributable to GN in WCBA across different regions

From the perspective of SDI regions, the ASIR of CKD attributable to GN in WCBA was highest in middle SDI regions, at 1.89 (95% UI, 2.73–1.16) per 1,00,000 population, with the highest number of cases (11,767; 95% UI, 16,836–7309). In high SDI regions, the ASIR was the lowest, at 1.1 (95% UI, 1.7–0.62) per 1,00,000 population, corresponding to a minimal number of reported cases (2869; 95% UI, 4325–1678). Across all SDI regions, the ASIR exhibited an upward trend, with the fastest growth rate observed in middle SDI countries, at 0.8 (95% UI, 0.77–0.82) per 1,00,000 population. Conversely, the lowest growth rate in ASIR was recorded in low SDI regions. With respect to the ASDR, the greatest number of cases were observed in middle SDI and low-middle SDI regions, at 3,75,911 (95% UI, 5,13,123–2,65,589) and 3,84,904 (95% UI, 5,13,920–2,74,930), respectively. In contrast, high SDI and high-middle SDI regions reported the fewest cases, with 86,141 (95% UI, 1,08,496–67,451) and 73,911 (95% UI, 98,258–53,349), respectively. In terms of growth trends, the fastest ASDR growth rate occurred in high SDI regions, at 1.23 (95% UI, 1.05–1.42) per 1,00,000 population, whereas negative growth was noted in upper-middle SDI and low SDI regions, with rates of −1.13 (95% UI, −1.32 to −0.94) per 1,00,000 population and −0.29 (95% UI, −0.47 to −0.11) per 1,00,000 population, respectively. These findings suggest that the severity and impact of CKD attributable to GN in WCBA are closely tied to local socioeconomic conditions.

**Figure 2.**
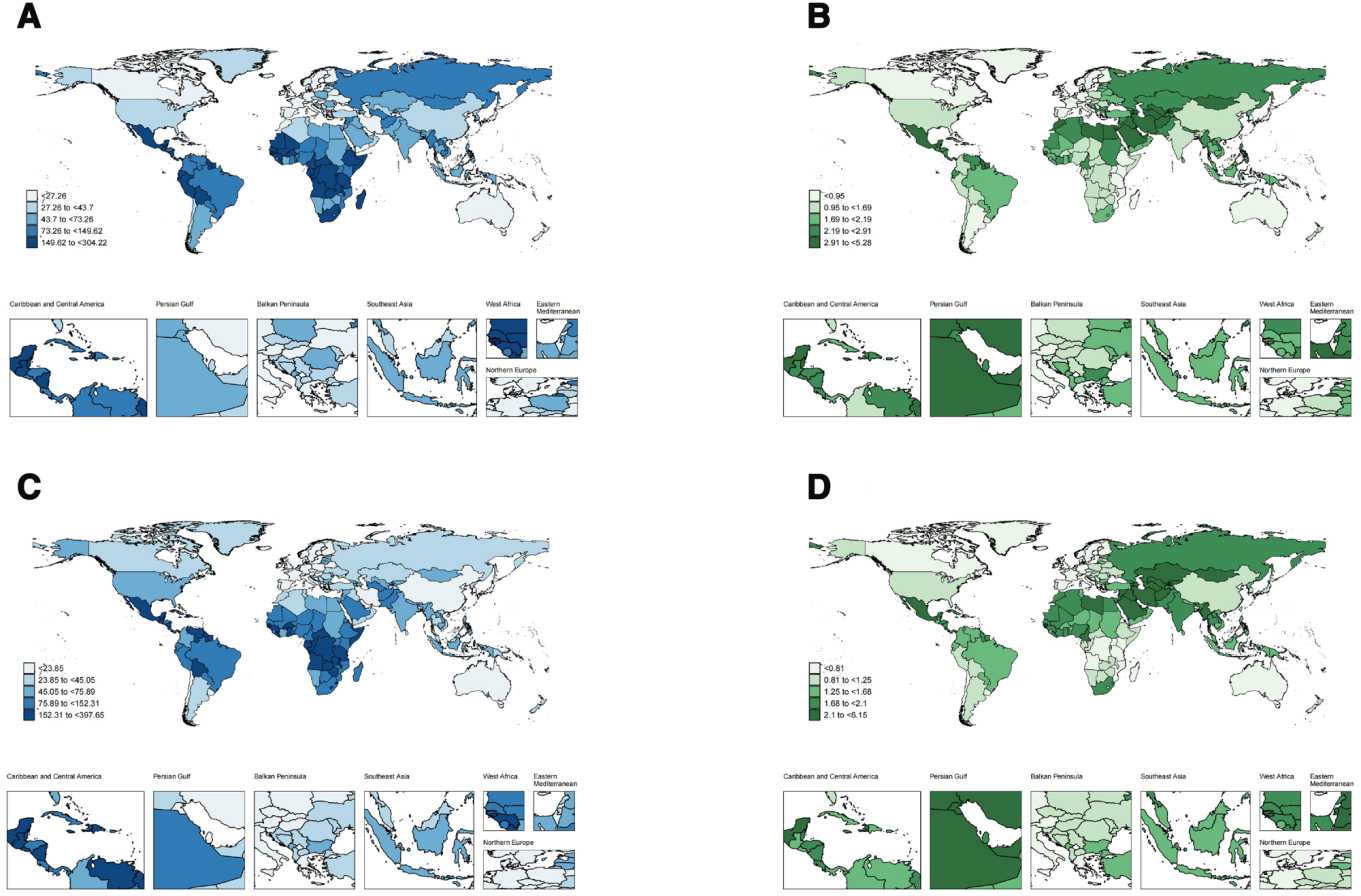
Global burden of CKD attributable to GN in WCBA by different nations in 1990 and 2021 (Table S3, Supplemental Digital Content 3). (A) ASDR in 1990, (B) ASIR in 1990, (C) ASDR in 2021, (D) ASIR in 2021. ASDR = age-standardized DALYs rate, ASIR = age-standardized incidence rate, CKD = chronic kidney disease, DALY = disability-adjusted life year, GN = glomerulonephritis, WCBA = women of childbearing age.

From the perspective of GBD regions, a comparative analysis revealed that the overall severity of the disease burden across different regions of the world remained largely stable from 1990 to 2021 (Fig. 2A−D, Tables 2, 3, S2 and S3, Supplemental Digital Content 2). According to the 2021 data, Central America exhibited the highest ASDR at 204.61 (95% UI, 282.55–140.76) per 1,00,000 population, while the high-income Asia-Pacific region had the lowest ASDR at 7.53 (95% UI, 10.32–5.18) per 1,00,000 population. In terms of ASIR, Central Asia had the highest at 3.43 (95% UI, 5.91–1.87) per 1,00,000 population, while Western Europe recorded the lowest at 0.64 (95% UI, 1.10–0.30) per 1,00,000 population. These regions should place greater emphasis on addressing the disease burden of CKD attributable to GN in WCBA.

**Table 2:** Cases, ASR and AAPC of CKD attributable to GN in WCBA by regions in 2021.

	Incidence					DALYs				
Regions	Case	ASR	95% UI	AAPC	95% UI	Case	ASR	95% UI	AAPC	95% UI
Andean Latin America	305	1.7	(64.25, 102.39)	1.36	(1.34, 1.38)	26,249	84.01	(0.99, 2.6)	−0.41	(−0.6, −0.22)
Australasia	65	1.7	(35.89, 77.1)	0.73	(0.71, 0.75)	1633	53.54	(0.99, 2.53)	0.36	(0.25, 0.45)
Caribbean	304	1.09	(47.1, 69.35)	1.43	(1.41, 1.45)	24,255	57.52	(0.58, 1.72)	1.15	(1.02, 1.25)
Central Asia	783	1.42	(16.61, 31.67)	0.8	(0.78, 0.83)	13,951	23.03	(0.75, 2.26)	0.74	(0.63, 0.87)
Central Europe	380	2.75	(140.74, 282.55)	0.92	(0.9, 0.93)	6623	204.61	(1.75, 3.92)	−2.18	(−2.29, −2.09)
Central Latin America	1884	0.84	(5.18, 10.32)	1.29	(1.26, 1.33)	1,41,288	7.53	(0.4, 1.43)	0.96	(0.83, 1.06)
Central Sub-Saharan Africa	349	3.43	(37.23, 81.65)	0.77	(0.75, 0.79)	54,573	56.52	(1.87, 5.91)	0.1	(0.03, 0.16)
East Asia	3647	1.06	(8.98, 21.44)	−0.13	(−0.15, −0.1)	49,804	14.49	(0.56, 1.67)	−2.06	(−2.11, −2.02)
Eastern Europe	1172	2.89	(28.55, 74.16)	1.19	(1.17, 1.21)	19,129	46.89	(1.72, 4.37)	−1.68	(−1.87, −1.46)
Eastern Sub-Saharan Africa	815	2.34	(26.08, 45.73)	0.07	(0.05, 0.09)	1,52,370	35.42	(1.32, 3.61)	−0.51	(−0.53, −0.48)
High-income Asia-Pacific	359	0.78	(103.17, 200.83)	−0.47	(−0.51, −0.44)	3233	148.15	(0.4, 1.24)	−2.85	(−2.95, −2.76)
High-income North America	973	2.54	(134.19, 324.07)	−0.21	(−0.26, −0.15)	51,594	200.19	(1.41, 4.14)	2.38	(2.31, 2.46)
North Africa and Middle East	4623	1.77	(102.43, 211.08)	1.26	(1.25, 1.28)	74,886	150.9	(0.88, 3.17)	0.17	(0.12, 0.22)
Oceania	79	2.11	(33.53, 83.95)	0.26	(0.25, 0.27)	1049	55.04	(1.25, 3.15)	0.55	(0.5, 0.59)
South Asia	8348	0.86	(14.95, 27.21)	0.08	(0.04, 0.12)	2,57,696	20.53	(0.34, 1.79)	0.09	(−0.01, 0.2)
Southeast Asia	3848	0.93	(20.2, 43.02)	0.92	(0.91, 0.94)	1,00,238	30.29	(0.41, 1.77)	−0.31	(−0.33, −0.29)
Southern Latin America	169	1.84	(77, 161.78)	0.58	(0.56 ,0.59)	5462	115.03	(1.06, 2.77)	−1.18	(−1.36, −0.98)
Southern Sub-Saharan Africa	377	1.07	(107.27, 270.68)	0.2	(0.18, 0.22)	28,698	175.8	(0.45, 2.02)	0	(−0.27, 0.28)
Tropical Latin America	1075	2.23	(17.46, 49.28)	0.38	(0.34, 0.4)	53,536	30.23	(1.12, 4.04)	−1.28	(−1.47, −1.16)
Western Europe	646	0.64	(10.28, 20.36)	0.15	(0.14, 0.15)	15,140	14.7	(0.3, 1.1)	−0.26	(−0.31, −0.2)
Western Sub-Saharan Africa	2210	1.74	(88.55, 192)	0.21	(0.2, 0.23)	1,30,057	133.52	(0.94, 2.72)	−0.4	(−0.45, −0.36)

AAPC = average annual percentage change, ASDR = age-standardized disability-adjusted life years rate, ASIR = age-standardized incidence rate, ASR = age-standardized rate, CKD = chronic kidney disease, DALY = disability-adjusted life year, GN = glomerulonephritis, SDI = Socio-demographic Index, WCBA = women of childbearing age.

**Table 3:** Cases, ASR and AAPC of CKD attributable to GN in WCBA by top 10 and bottom 10 countries in 2021.

	Incidence					DALYs				
Countries	CASE	ASIR	95% UI	AAPC	95% UI	CASE	ASDR	95% UI	AAPC	95% UI
Bahamas	3	2.87	(1.11, 6.05)	1.25	(1.23, 1.26)	313	285.4	(181.92, 411.66)	1.26	(1.04, 1.44)
Belize	4	3.4	(1.29, 7.07)	1.74	(1.7, 1.77)	364	311.71	(216.74, 424.79)	1.79	(1.49, 2.06)
Congo	24	1.63	(0.56, 3.57)	0.86	(0.84, 0.88)	3894	279.97	(148.63, 479.14)	0.66	(0.49, 0.87)
Democratic Republic of the Congo	218	1.03	(0.34, 2.23)	0.75	(0.73, 0.77)	34,780	171.91	(95.65, 282.41)	0.03	(−0.08, 0.14)
Denmark	9	0.67	(0.21, 1.51)	0.21	(0.19, 0.22)	177	13.1	(7.27, 22.63)	0.01	(−0.07, 0.07)
Dominica	1	3.4	(1.26, 7.09)	0.94	(0.91, 0.97)	45	276.25	(171.41, 414.09)	1.98	(1.91, 2.04)
Dominican Republic	70	2.42	(0.94, 5.16)	2.08	(2.05, 2.11)	5790	200.78	(127.57, 293.29)	1.22	(1.09, 1.34)
El Salvador	47	2.67	(1.02, 5.72)	1.7	(1.66, 1.75)	4930	279.38	(167.54, 428.35)	2.37	(2.1, 2.63)
Finland	8	0.68	(0.22, 1.49)	1.31	(1.27, 1.34)	128	10.4	(5.26, 18.75)	0.18	(0.12, 0.24)
France	106	0.69	(0.21, 1.64)	0.54	(0.52, 0.56)	1488	9.62	(6.41, 14.01)	−0.26	(−0.32, −0.21)
Grenada	1	3.49	(1.3, 7.63)	1.65	(1.63, 1.67)	85	332.98	(227.76, 460.34)	0.36	(0.1, 0.54)
Guyana	6	3.21	(1.21, 6.84)	1.6	(1.58, 1.62)	796	397.65	(237.96, 602.65)	2.41	(2.14, 2.69)
Iceland	0	0.56	(0.17, 1.31)	−0.19	(−0.2, −0.17)	9	10.48	(6.39, 17.28)	0.73	(0.67, 0.78)
Japan	246	0.89	(0.43, 1.51)	−0.28	(−0.34, −0.23)	1063	3.72	(2.26, 5.66)	−0.52	(−0.55, −0.49)
Netherlands	22	0.55	(0.17, 1.27)	0.53	(0.51, 0.54)	526	13.42	(8.49 ,20.95)	−0.15	(−0.22, −0.08)
Norway	7	0.51	(0.21, 0.95)	0.41	(0.38, 0.43)	144	11.01	(7.29, 15.99)	0.26	(0.2, 0.32)
Suriname	4	2.93	(1.11, 6.25)	1.37	(1.35, 1.4)	484	330.13	(206.94, 491.67)	2.05	(1.78, 2.4)
Sweden	10	0.42	(0.15, 0.9)	−0.54	(−0.56, −0.51)	262	10.93	(6.96, 16.42)	0.49	(0.41, 0.57)
Saint Vincent and the Grenadines	1	3.06	(1.19, 6.53)	1.36	(1.34, 1.39)	93	326.93	(223.05, 456.07)	1.69	(1.36, 1.93)
Republic of Korea	92	0.71	(0.24, 1.54)	−0.97	(−1.01, −0.93)	1719	13.23	(8.75, 19.02)	−4.39	(−4.57, −4.24)

AAPC = average annual percentage change, ASDR = age-standardized disability-adjusted life years rate.(Full country‑level estimates are provided in Table S1, Supplemental Digital Content 1), ASIR = age-standardized incidence rate, ASR = age-standardized rate, CKD = chronic kidney disease, DALY = disability-adjusted life year, GN = glomerulonephritis, SDI = Socio-demographic Index, WCBA = women of childbearing age.

When considering specific countries/territories, those with the highest disease burden warrant particular attention. The top 3 countries with the highest ASDR were Guyana, Grenada, and Suriname. Conversely, the 3 countries with the lowest ASDR were Japan, France, and Finland. The highest ASIR values were observed in the Solomon Islands, the Northern Mariana Islands, and Micronesia. The lowest ASIR, however, was recorded in Sweden, Spain, and Norway. Over the past 31 years, the 3 countries with the fastest growth rates were Ukraine, Lesotho, and Zimbabwe, whereas the countries with the steepest negative growth rates were South Korea, Poland, and Romania.

### 3.3. The relationship between the disease burden in CKD attributable to GN in WCBA and SDI, age, and year

**Figure 3.**
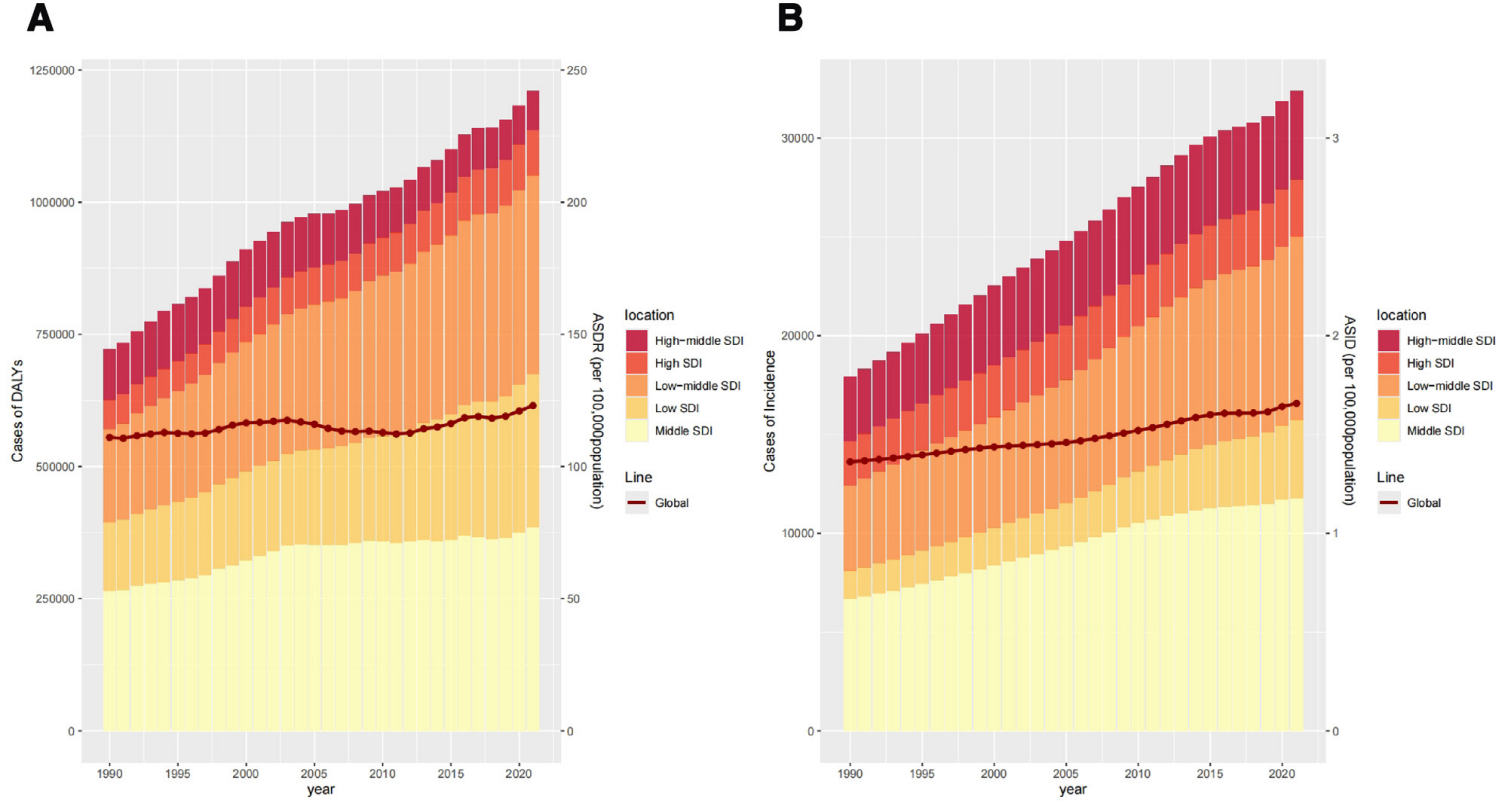
ASDR and ASIR of CKD attributable to GN in WCBA by different SDI regions and years globally. (A) ASDR, (B) ASIR. ASDR = age-standardized disability-adjusted life years rate, ASIR = age-standardized incidence rate, CKD = chronic kidney disease, DALY = disability-adjusted life year, GN = glomerulonephritis, SDI = Socio-demographic Index, WCBA = women of childbearing age.

This study analyzed the proportions of disease burden across different SDI regions over various years and age groups. Based on the analysis of ASDR, the global ASDR remained relatively stable over the past 31 years (Fig. 3A). In terms of total DALYs, the middle SDI region exhibited the highest proportion, although this share gradually declined over time, accompanied by a similar decrease in the high SDI and high-middle SDI regions. This trend may be attributed to improvements in socioeconomic development and healthcare infrastructure. Notably, the proportion of ASDR in low SDI and low-middle SDI regions increased year by year, suggesting that these regions may be experiencing a rising disease burden.

From the perspective of age groups, we observed a steady increase in ASDR with advancing age. In the 15 to 19 and 20 to 24 age groups, the proportion of ASDR in low SDI regions was the highest. However, as age increased, the proportion of ASDR in low SDI regions gradually decreased, while the proportions in high SDI and high-middle SDI regions increased. This trend suggests that, with increasing economic development, the incidence of CKD in WCBA due to GN in younger age groups may have been better managed and treated. These findings underscore the need for greater focus on monitoring and treating younger WCBA with GN and CKD in lower SDI regions (Fig. 3B).

**Figure 4.**
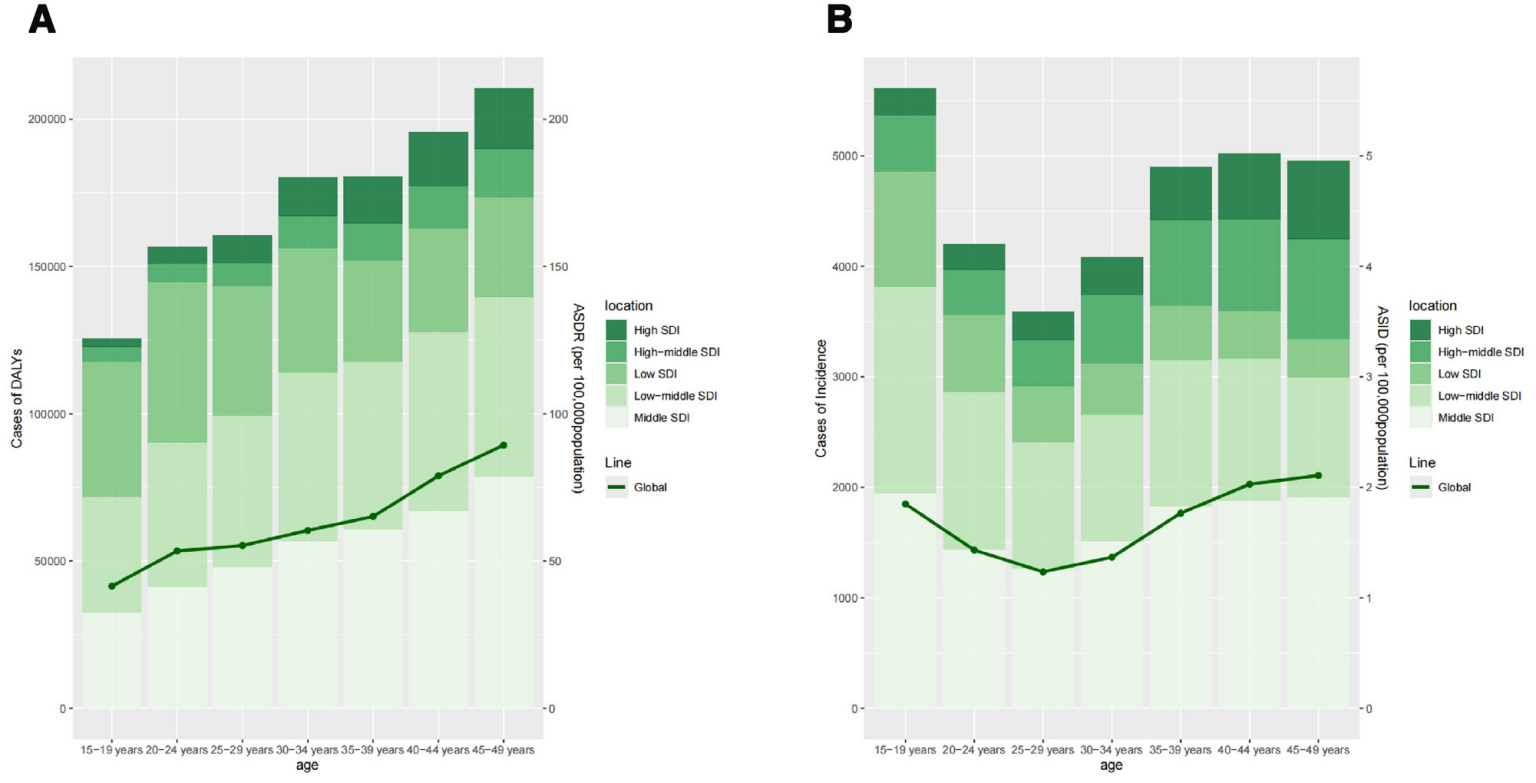
ASDR and ASIR of CKD attributable to GN in WCBA by different nations in 1990 and 2021 by different SDI regions and ages in 1990. Notes: (A) ASDR, (B) ASIR. ASDR = age-standardized DALYs rate; ASIR = age-standardized incidence rate, CKD = chronic kidney disease, DALY = disability-adjusted life year, GN = glomerulonephritis, SDI = Socio-demographic Index, WCBA = women of childbearing age.

Over the past 31 years, the ASIR showed a steady but modest increase. In terms of SDI composition, the number of cases in middle SDI regions remained the highest throughout the period, while the number of cases in high SDI regions consistently remained the lowest. However, the proportion of ASIR in low SDI and low-middle SDI regions increased annually, while the number of cases in high SDI and high-middle SDI regions gradually declined. This trend mirrored the ASDR results, indicating that, as economic development improved, the disease burden of CKD attributable to GN in WCBA progressively decreased (Fig. 4A).

From the perspective of age groups, the global incidence rate followed a U-shaped distribution, with the lowest ASIR observed in the 25 to 29 age group and the highest rates in the 40 to 44 and 45 to 49 age groups, followed by the 15 to 19 age group. Regarding SDI region composition, as age increased, the proportion of cases in low SDI and lower-middle SDI regions gradually decreased, while the proportion in high SDI and upper-middle SDI regions increased. This pattern was consistent with the ASDR findings, suggesting that, with increasing economic development, the average age of onset for CKD in WCBA due to GN gradually shifted to older age groups. This indicates that low SDI regions should place more emphasis on the disease burden in younger WCBA, while high SDI regions should allocate more resources to the care of older WCBA (Fig. 4B).

### 3.4. Frontier analysis of the relationship between the burden of CKD attributable to GN in WCBA and SDI

**Figure 5.**
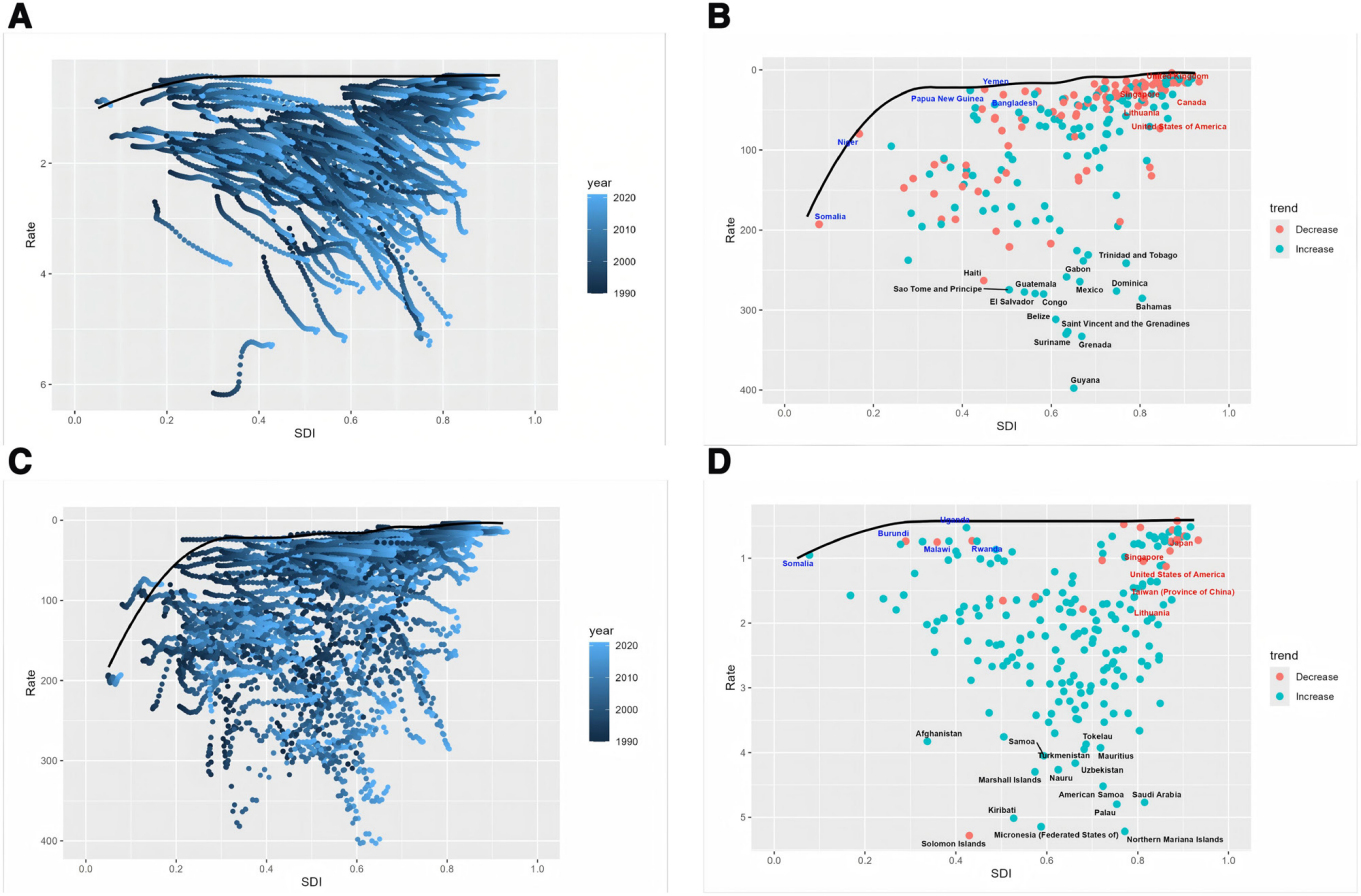
Frontier analysis based on SDI and disease burden of CKD attributable to GN in WCBA in 204 countries and territories. (A and B) ASDR of CKD attributable to GN in WCBA, (C and D) ASIR of CKD attributable to GN in WCBA. ASDR = age-standardized disability-adjusted life years rate, ASIR = age-standardized incidence rate, CKD = chronic kidney disease, DALY = disability-adjusted life year, GN = glomerulonephritis, SDI = Socio-demographic Index, WCBA = women of childbearing age.

A frontier analysis was conducted to explore the optimal achievable disease burden management for CKD attributable to GN in WCBA at each country’s specific SDI level. Countries located farther from the fitted frontier line indicate a potential underperformance in controlling the disease burden given their current SDI, suggesting a need for increased investment or more effective policies. Conversely, countries closer to the frontier represent better-performing systems whose policies and prevention/management approaches could serve as valuable references for other nations at similar SDI levels. Regarding the ASDR, the countries furthest from the frontier line in WCBA with CKD attributable to GN included Guyana, Suriname, Grenada, Saint Vincent and the Grenadines, Belize, the Bahamas, and others. In high-SDI countries, the 5 countries closest to the fitted line were the United Kingdom, Singapore, Canada, the Republic of Lithuania, and the United States. In low-SDI countries, the 5 countries closest to the frontier line were Yemen, Papua New Guinea, Bangladesh, Niger, and Somalia (Fig. 5A, B).

In terms of ASIR, the countries furthest from the frontier line with CKD attributable to GN in WCBA included the Solomon Islands, the Commonwealth of the Northern Mariana Islands, the Federated States of Micronesia, the Republic of Kiribati, Palau, American Samoa, Saudi Arabia, and others. In high SDI countries, the 5 nations closest to the fitted line were Japan, Singapore, the United States, Taiwan, and the Republic of Lithuania. In low SDI countries, the 5 countries closest to the frontier line were Uganda, Burundi, the Republic of Mawila, Rwanda, and Somalia (Fig. 5C, D).

### 3.5. Health inequality analysis

**Figure 6.**
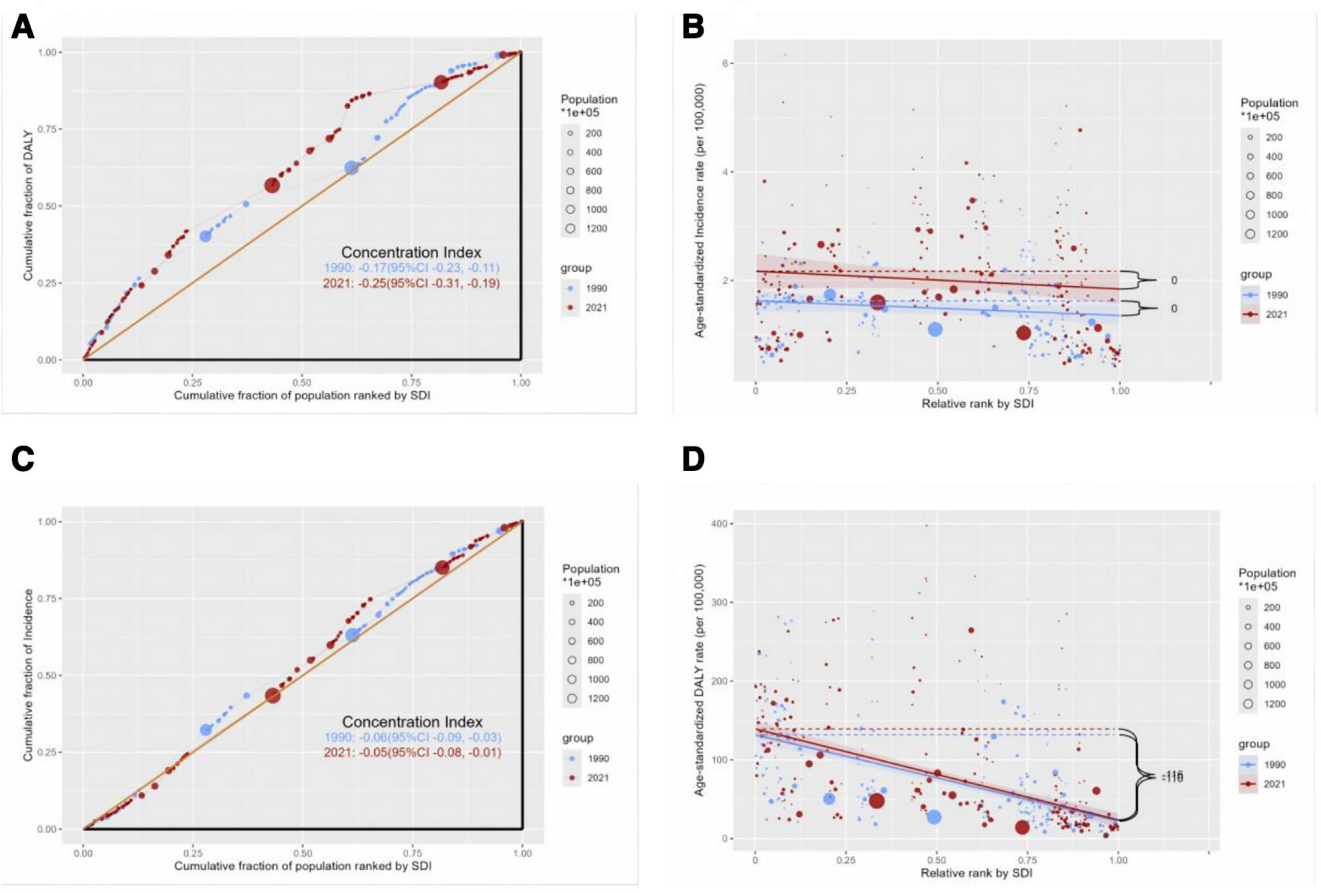
Slope indexes and concentration indexes for CKD attributable to GN in WCBA from 1990 to 2021 worldwide. (A) ASDR of CKD attributable to GN in WCBA by SDI, (B) ASIR of CKD attributable to GN in WCBA by SDI. ASDR = age-standardized DALYs rate; ASIR = age-standardized incidence rate, CKD = chronic kidney disease, DALY = disability-adjusted life year, GN = glomerulonephritis, SDI = Socio-demographic Index, WCBA = women of childbearing age.

Building on the previous conclusion, the study found a strong correlation between the ASIR and ASDR of CKD attributable to GN in WCBA and the SDI. Further analysis of health inequalities is presented. The slope index for ASDR slightly decreased from −108 (95% UI: −132.07 to −83.92) per 1,00,000 population in 1990 to −109.05 (95% UI: −139.34 to −78.76) per 1,00,000 population in 2021, indicating a downward trend in ASDR with the continuous increase in SDI. However, the change in the slope between 1990 and 2021 was not statistically significant, suggesting that health inequalities persisted and remained relatively unchanged over time. The concentration index for ASDR slightly decreased from −0.17 (95% UI: −0.11 to −0.23) per 1,00,000 population in 2019 to −0.25 (95% UI: −0.19 to −0.31) per 1,00,000 population in 2021. These findings suggest that regions with higher SDI tend to have lower ASDR, with larger absolute values, highlighting pronounced health inequities (Fig. 6A).

For ASIR, the slope index decreased from −0.28 (95% UI: −0.61 to 0.04) per 1,00,000 population in 1990 to −0.32 (95% UI: −0.77 to 0.13) per 1,00,000 population in 2021, while the concentration index slightly increased from −0.06 (95% UI: −0.03 to −0.09) per 1,00,000 population in 1990 to −0.05 (95% UI: −0.01 to −0.08) per 1,00,000 population in 2021. Therefore, health inequality did not significantly affect ASIR but had a considerable impact on ASDR. These findings suggest that regions with higher levels of economic development tend to provide better management and care conditions for WCBA, though such conditions have not been as effective in reducing the incidence of CKD (Fig. 6B).

### 3.6. Projections of the disease burden of CKD attributable to GN in WCBA up to 2050

**Figure 7.**
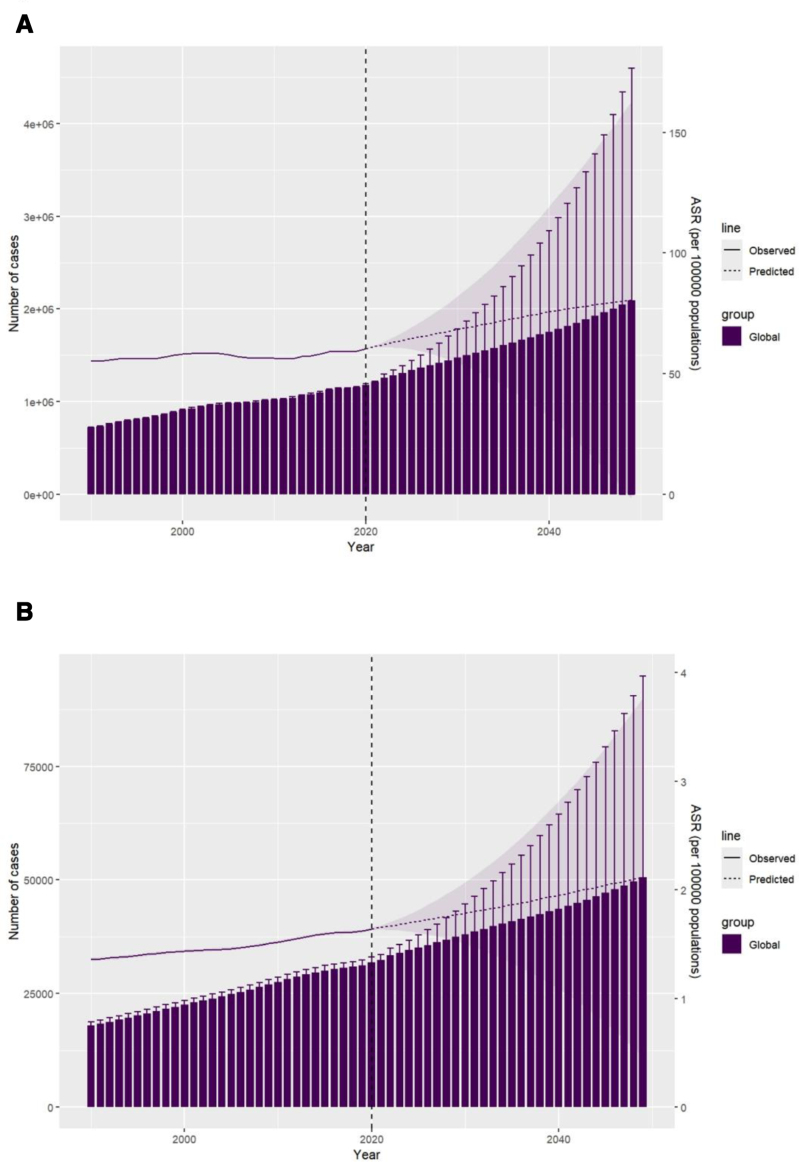
Overall projection of CKD attributable to GN in WCBA from 2022 to 2049. (A) ASDR of CKD attributable to GN in WCBA, (B) ASIR of CKD attributable to GN in WCBA. ASDR = age-standardized disability-adjusted life years rate, ASIR = age-standardized incidence rate, CKD = chronic kidney disease, DALY = disability-adjusted life year, GN = glomerulonephritis, WCBA = women of childbearing age.

**Figure 8.**
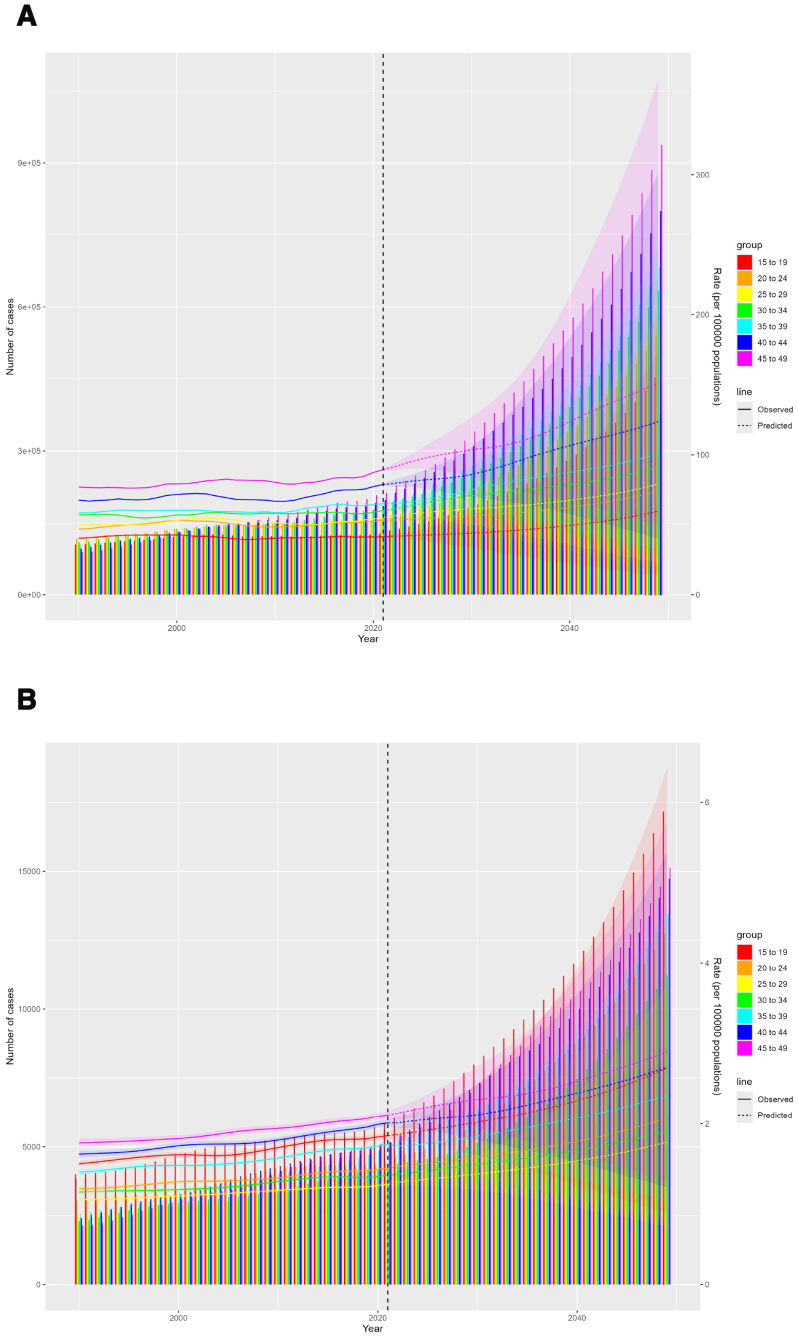
Projection of CKD attributable to GN in WCBA by different age groups from 2022 to 2049. (A) ASDR by age groups, (B) ASIR by age groups. ASDR = age-standardized DALYs rate; ASIR = age-standardized incidence rate, CKD = chronic kidney disease, DALY = disability-adjusted life year, GN = glomerulonephritis, WCBA = women of childbearing age.

In this study, the incidence and DALYs of CKD attributable to GN in WCBA over the next 28 years (2022–2049) were projected by integrating anticipated demographic changes with the BAPC model. The results suggested a global increase in both the incidence and DALYs of CKD attributable to GN in WCBA. The prediction model indicates that, from 2022 to 2049, global incidence and DALYs are expected to rise to 46,580 (95% UI, 9921–83,238) and 17,62,263 (95% UI, −42,478 to 35,37,552), respectively. The ASIR and ASDR are projected to increase to 2.12 (95% UI, 0.46–3.77) per 1,00,000 population and 80.51 (95% UI, −1.96 to 162.97) per 1,00,000 population, respectively (Fig. 7). When stratified by age group, the highest incidence and DALYs were observed in the 45 to 49 age group, with values of 2.89 (95% UI, 1.29–5.75) per 1,00,000 population and 151.08 (95% UI, 49.04–368.42) per 1,00,000 population, respectively (Fig. 8, Table 4, Table S2, Supplemental Digital Content 2).

**Table 4:** Projection of CKD attributable to GN in WCBA by age groups from 2022 to 2049.

Forecast by age group of ASDR from 2022 to 2049																												
Age groups	2022	2023	2024	2025	2026	2027	2028	2029	2030	2031	2032	2033	2034	2035	2036	2037	2038	2039	2040	2041	2042	2043	2044	2045	2046	2047	2048	2049
15–19	41.84	42.08	42.33	42.59	42.86	43.14	43.45	43.77	44.12	44.49	44.89	45.33	45.79	46.30	46.84	47.43	48.07	48.76	49.51	50.33	51.21	52.16	53.20	54.32	55.54	56.86	58.30	59.86
20–24	53.47	53.89	54.27	54.61	54.94	55.28	55.64	56.02	56.43	56.86	57.32	57.82	58.35	58.93	59.55	60.22	60.94	61.73	62.58	63.50	64.50	65.58	66.75	68.02	69.40	70.89	72.52	74.28
25–29	56.42	57.26	58.02	58.72	59.37	59.95	60.47	60.95	61.40	61.85	62.33	62.83	63.37	63.95	64.58	65.25	65.97	66.75	67.59	68.50	69.48	70.55	71.70	72.95	74.30	75.77	77.36	79.08
30–34	61.28	62.55	63.92	65.32	66.68	67.89	68.96	69.94	70.86	71.74	72.54	73.29	74.00	74.69	75.39	76.14	76.94	77.80	78.73	79.73	80.80	81.96	83.21	84.57	86.03	87.61	89.32	91.17
35–39	65.53	66.00	66.68	67.63	68.82	70.17	71.68	73.33	75.02	76.67	78.18	79.54	80.81	82.03	83.22	84.34	85.41	86.46	87.51	88.59	89.74	90.98	92.32	93.75	95.30	96.96	98.76	100.70
40–44	79.57	80.47	81.30	82.00	82.56	83.08	83.75	84.69	85.99	87.62	89.47	91.54	93.80	96.15	98.47	100.63	102.63	104.54	106.41	108.27	110.06	111.82	113.59	115.38	117.24	119.24	121.39	123.70
45–49	91.14	93.31	95.35	97.20	98.78	100.12	101.34	102.48	103.48	104.31	105.12	106.14	107.52	109.38	111.68	114.29	117.22	120.43	123.78	127.14	130.33	133.35	136.30	139.24	142.20	145.14	148.08	151.08
Forecast by age group of ASIR from 2022 to 2049																												
age groups	2022	2023	2024	2025	2026	2027	2028	2029	2030	2031	2032	2033	2034	2035	2036	2037	2038	2039	2040	2041	2042	2043	2044	2045	2046	2047	2048	2049
15–19	1.87	1.88	1.90	1.92	1.94	1.96	1.98	2.00	2.02	2.04	2.06	2.09	2.11	2.14	2.16	2.19	2.22	2.25	2.29	2.32	2.36	2.40	2.44	2.49	2.53	2.58	2.64	2.69
20–24	1.47	1.48	1.50	1.51	1.52	1.54	1.55	1.57	1.58	1.60	1.62	1.63	1.65	1.67	1.69	1.71	1.73	1.76	1.78	1.81	1.83	1.86	1.89	1.92	1.96	1.99	2.03	2.07
25–29	1.27	1.29	1.30	1.31	1.32	1.34	1.35	1.36	1.38	1.39	1.40	1.42	1.43	1.45	1.47	1.48	1.50	1.52	1.54	1.56	1.58	1.60	1.63	1.65	1.68	1.71	1.74	1.77
30–34	1.37	1.38	1.40	1.42	1.43	1.45	1.46	1.48	1.49	1.51	1.52	1.54	1.56	1.57	1.59	1.61	1.63	1.65	1.67	1.69	1.71	1.74	1.76	1.79	1.81	1.84	1.87	1.91
35–39	1.72	1.73	1.74	1.75	1.77	1.78	1.80	1.82	1.84	1.87	1.89	1.91	1.93	1.95	1.98	2.00	2.02	2.05	2.07	2.10	2.12	2.15	2.18	2.21	2.25	2.28	2.32	2.36
40–44	2.01	2.03	2.04	2.05	2.06	2.07	2.08	2.10	2.11	2.13	2.15	2.18	2.20	2.23	2.26	2.29	2.32	2.35	2.38	2.41	2.44	2.48	2.51	2.54	2.58	2.62	2.66	2.70
45–49	2.13	2.16	2.18	2.21	2.23	2.25	2.27	2.29	2.30	2.31	2.33	2.34	2.36	2.38	2.40	2.43	2.46	2.49	2.53	2.56	2.60	2.64	2.68	2.72	2.76	2.80	2.84	2.89

ASDR = age-standardized disability-adjusted life years rate, ASIR = age-standardized incidence rate, CKD = chronic kidney disease, DALY = disability-adjusted life year, GN = glomerulonephritis, SDI = Socio-demographic Index, WCBA = women of childbearing age.

## 4. Discussion

This study provides a comprehensive analysis and prediction of CKD attributable to GN in WCBA. The analysis spanned from 1990 to 2021 and incorporated various factors, including ages, countries, and regions. Over this 32-year period, the disease burden of CKD attributable to GN in WCBA has significantly increased. The sustained increase in disease burden can be attributed to several factors: population growth and shifting age structures within WCBA, improved detection and reporting systems in certain regions, the persistent presence of infection-related GN among vulnerable populations, and uneven coverage of effective interventions (including early diagnosis, access to renal biopsy and immunosuppressive therapy, and long-term CKD management). Inadequate attention to WCBA as a distinct population group, coupled with insufficient recognition of their substantial disease burden, may explain the current lack of effective intervention measures. This oversight highlights the urgent need for targeted health policies that address the specific epidemiological characteristics and healthcare disparities affecting this demographic. Previous epidemiological studies on CKD in women have primarily focused on all age groups or CKD caused by various factors, without specifically analyzing certain subgroups or disease causes.^[18,19]^ This limitation impedes the assessment of the disease burden of CKD attributable to GN in the vulnerable population of WCBA. To address this gap, the current study aimed to bring attention to this specific group through targeted analysis and offered deeper insights into the health impacts of CKD attributable to GN. Several studies have suggested a correlation between CKD and physiological changes in WCBA.^[20]^ Advanced stages of CKD are characterized by renal failure, proteinuria, hypertension, and poor control of underlying primary kidney diseases – all significant risk factors for adverse pregnancy and renal outcomes.^[21]^ During pregnancy, women undergo substantial changes in blood volume, blood pressure, and blood viscosity to support fetal growth and development, which increases the renal burden. Research has indicated that a decline in estimated glomerular filtration rate and an increase in urinary protein are associated with the deterioration of renal function during pregnancy.^[22]^ Pregnancy-related GN may manifest as acute kidney injury or as an exacerbation of preexisting CKD. In some countries, influenced by socio-cultural norms and economic conditions, early marriage and childbirth are prevalent, exposing young girls to higher health risks, including mental health issues.^[23]^ Gender inequality persists in many regions, which can create barriers for women in sexual relationships, potentially increasing their vulnerability to infections and diseases.^[24,25]^ In low- and middle-income settings, women often face limited access to healthcare and lower treatment rates for CKD. Social norms, medical stereotypes, and caregiving responsibilities can further impede women’s access to nephrology care compared to men.^[26]^ Studies indicate that pregnant women with CKD are less likely to be referred to nephrologists and receive less intensive CKD management compared to men, potentially worsening disease outcomes and burden in WCBA.^[27,28]^ In addition, research indicates that women with CKD at various stages face increased renal and reproductive health risks during pregnancy.^[29]^ Furthermore, pregnancies in women with CKD are associated with higher rates of adverse fetal outcomes.^[30]^ For older mothers (women over 35) with CKD, the risk of adverse pregnancy outcomes is further elevated.^[31]^ These complications contribute significantly to the broader economic costs. These findings underscore the need for in-depth, targeted research on kidney diseases like CKD and GN in the WCBA population.

From the perspective of ASDR, the highest rates were observed in low-middle and middle SDI regions, while the lowest rates were found in high and high-middle SDI regions. This discrepancy is likely attributed to more effective management policies for high-risk factors, such as glomerular diseases and hypertension, in higher SDI regions. In contrast, countries with lower SDI levels often lack adequate healthcare policies for CKD attributable to GN and face challenges related to poorer health services. Consequently, infection-related GN remains a major cause of CKD in many impoverished and developing areas.^[32]^ Research indicates that high-SDI regions tend to invest more in healthcare systems, infrastructure, sanitation, and medical resources, which often translates to a greater healthcare workforce capacity that can mitigate the progression of CKD.^[33]^ A study on RRT across 67 European countries suggested that countries with relatively lower SDI had significantly fewer facilities offering such treatment. Importantly, the lower incidence of CKD attributable to GN in these countries was not maintained; rather, due to limitations in treatment capacity, the burden of CKD attributable to GN in WCBA may be exacerbated.^[34]^ These regions require heightened attention to disease management and treatment for WCBA, as well as significant improvements in healthcare infrastructure.^[35,36]^ Furthermore, global collaboration should be intensified, with increased focus on low-income countries, ensuring these regions gain access to more funding and advanced diagnostic and therapeutic technologies. Specifically, we note that the lower ASIR and ASDR of CKD attributable to GN among WCBA in low-SDI regions may be partially attributable to poorer quality of vital registration systems in these areas, potentially leading to underreporting of the CKD attributable to GN burden. Furthermore, limitations in medical technology and diagnostic capacity in low-SDI regions might result in lower detection rates for CKD attributable to GN, further impacting case reporting. Therefore, enhancing medical technology, diagnostic capabilities, and establishing more robust vital registration systems in these countries are crucial for obtaining a more accurate assessment of the CKD attributable to GN burden among WCBA.

Geographically, the disease burden is relatively lighter in Western Europe and high-income Central Asia, while it is more pronounced in Central America and the Asia-Pacific region. This disparity may be attributed to factors such as more stable social development, rapid improvements in healthcare resources, and heightened public awareness of the disease.^[37]^ A study indicates that by 2019, approximately 40 million individuals in Western Europe were affected by CKD,^[38]^ with renal replacement therapy (RRT) alone accounting for approximately 2% of healthcare expenditures in the region. This underscores the significant attention that Western European countries devote to CKD. In comparison to other regions, Western Europe boasts a more robust healthcare infrastructure, with extensive coverage of essential resources such as healthcare professionals, medications, and universal health insurance.^[39]^ This comprehensive system enables more effective alleviation of the disease burden. For instance, in the United Kingdom, during 2009 to 2010, the National Health Service in England allocated approximately £1.45 billion for the treatment of CKD patients, which represented around 1.3% of its total budget.^[40]^ The UK government has introduced the Quality and Outcomes Framework for General Practice (QOF) to incentivize the early identification and management of conditions such as CKD and hypertension. Guidelines have been established to recommend diagnostic and treatment pathways for CKD, facilitating early diagnosis and timely intervention. In high-income Central Asia, Kazakhstan serves as an example where citizens benefit from full government subsidies for RRT. The National Cardiovascular Disease Management Healthcare Program, launched in Kazakhstan in 2005, has substantially improved life expectancy among CKD patients.^[41]^ Following the implementation of healthcare policies in 2010, there has been a marked increase in dialysis opportunities for patients in remote and underserved areas. Furthermore, Kazakhstan is committed to enhancing organ transplantation services, with its deceased donor program experiencing rapid expansion.^[42]^ Through a series of healthcare reforms, improved treatment options for CKD patients have been made available, significantly alleviating the burden of CKD in Kazakhstan. The world map of disease burden indicates that the Australian region has a relatively low and stable burden of CKD attributable to GN in WCBA over the years. This favorable outcome may be partly linked to the mandatory notification of GN for clinicians established in Australia in 1991, alongside the creation of a GN registry to enhance accurate disease surveillance and public health responses.^[11]^ This policy initiative appears to have contributed significantly to reducing the burden of CKD attributable to GN. These examples underscore how appropriate kidney health strategies or specific interventions can effectively lower the burden of CKD attributable to GN in WCBA.

In contrast, research suggests that many countries in the Central America and Asia-Pacific region face challenges in providing kidney replacement therapy for CKD,^[11]^ including insufficient renal care capacity, limited public funding, inadequate research capabilities, and a scarcity of opportunities for kidney replacement therapy. For example, in South Asia, several countries experience slow economic development and low government spending on health, which has weakened renal care capacity and perpetuated misconceptions about renal care, thereby exacerbating the burden of CKD.^[43]^ As of 2020, statistical estimates show that the number of CKD patients in India reached approximately 140.2 million.^[44]^ Coupled with the country’s aging population, the burden of CKD is becoming increasingly severe. In India, there is a lack of systematic data collection and management for CKD patients, and existing healthcare services and policies struggle to support patients in accessing low-cost and effective treatment options.^[45]^ To address the significant burden of CKD, some countries in the Asia-Pacific region have begun offering RRT, and India has gradually initiated substantial subsidies or free programs for RRT.^[4,5,46,47]^ However, challenges such as a shortage of specialized healthcare personnel and limited program coverage persist.

Analysis of the disease burden across different age groups revealed that the 15 to 19 age group had the highest number of incident cases, yet the peak ASIR was observed in the 45 to 49 age group. This discrepancy may be attributed to the higher incidence of infection-triggered GN in individuals under 20,^[48]^ leading to the progression of CKD from GN in WCBA. However, the relatively smaller population size in the 45 to 49 age group likely contributes to its higher ASIR compared to the 15 to 19 group. Concurrently, the highest ASDR was observed in the 45 to 49 age group. This may be because although the incidence of CKD attributable to GN is higher in the 15 to 19 age group, better physical condition and earlier intervention likely lead to a more favorable prognosis and lower DALYs. After age 25, the incidence of infection-triggered GN gradually decreases. However, with advancing age, the prevalence of GN and CKD rises again due to factors like diabetes, hypertension, cardiovascular disease, advanced age, obesity, and physical inactivity. The risks associated with older age also contribute to the highest ASDR in the 45 to 49 group. Furthermore, with increasing age, CKD may progress to end-stage renal disease. In resource-limited settings, limited access to RRT (dialysis, transplantation) further elevates ASDR. Consequently, cases originating in adolescence, if not effectively treated, may reach their terminal stage in middle age, manifesting as premature mortality and severe disability due to end-stage renal disease and its cardiovascular complications, thereby increasing DALYs. This pattern reveals that in regions with weaker healthcare systems, the true burden of chronic diseases like CKD attributable to GN is reflected in late-stage complications and mortality (DALYs) rather than incidence alone, resulting in a “U”-shaped trend. This underscores the need for targeted strategies: promoting infection treatment and health education for younger WCBA to reduce exposure, and encouraging regular health assessments for older WCBA to prevent metabolic diseases. Government policies facilitating regular health checkups in this age group are crucial to alleviate the CKD attributable to GN burden.

From the perspective of growth rates, the disease burden of CKD attributable to GN in WCBA across nearly all age groups exhibited a rapid increase from 2019 to 2021, potentially linked to the COVID-19 pandemic.^[49]^ This observed surge may be significantly associated with the COVID-19 pandemic. Research indicates that CKD patients infected with COVID-19 face a substantially higher risk of rapid deterioration in estimated glomerular filtration rate, showing a 3.7-fold increase compared to noninfected patients.^[50]^ Furthermore, patients with chronic kidney disease are more susceptible to COVID-19, and evidence suggests that COVID-19 is associated with severe kidney damage, consequently leading to an increased mortality risk.^[51,52]^ Simultaneously, the COVID-19 pandemic has caused massive disruptions to global healthcare systems. Beyond the acute phase, care visits for CKD patients plummeted dramatically, which has further exacerbated the burden of CKD attributable to GN in WCBA.^[53]^ Additionally, pregnancy itself is a recognized high-risk factor for severe COVID-19 outcomes.^[54,55]^ Thus, under the influence of the pandemic,^[56]^ the CKD disease burden attributable to GN in WCBA has further escalated. Consequently, we advocate for nations to establish more resilient healthcare systems capable of withstanding such unforeseen global events. Our future research will further investigate the burden of kidney injury associated with COVID-19 and assess the impact of these emerging risk factors on CKD due to GN in the WCBA population. This content has been added to the Discussion section.

Currently, a variety of treatment strategies have been developed for CKD and GN, including the use of angiotensin-converting enzyme inhibitors and angiotensin receptor blockers.^[57]^ Dialysis remains the primary treatment modality for patients with advanced CKD who are not suitable candidates for or are less likely to undergo preemptive kidney transplantation.^[58-60]^ However, there is still a research gap regarding the specific measures that can be taken for WCBA when facing GN and CKD. Therefore, it is imperative that governments and healthcare institutions allocate more attention and resources to WCBA, conduct in-depth research, develop novel pharmacological interventions, and adopt more advanced therapeutic strategies to address CKD resulting from GN. Based on the above, our findings suggest that WCBA represent a vulnerable group, with the burden of CKD attributable to GN increasing annually. It is also essential to recognize that patient management plans should be tailored to the specific characteristics of different regions and age groups. This study may serve as a reference for government departments in formulating healthcare policies for WCBA. Additionally, it offers potential research directions for medical professionals.

This study has limitations despite findings on CKD attributable to GN in WCBA. First, data quality and scope in GBD 2021 may affect accuracy, leading to underreporting or misdiagnosis, especially in low-SDI regions. Moreover, unmeasured confounding factors may influence comparisons across different SDI regions. Furthermore, the data within GBD 2021 constitute aggregated data, lacking detailed information at the individual patient level. This includes specifics on patient comorbidities, underlying health conditions (such as diabetes, hypertension, and other diseases potentially contributing to CKD), reproductive health exposure factors (including preeclampsia, infections, and high-risk pregnancies), as well as upstream environmental determinants (including access to clean water and exposure to environmental nephrotoxins such as heavy metals and pesticides). Consequently, the analysis of these specific risk factors in relation to CKD burden is precluded. Future research utilizing other databases containing individual-level information is warranted to delve deeper into the individualized attributions and interactions of comorbidities in CKD attributable to GN among WCBA. It is important to note that other data sources, such as those from the International Agency for Research on Cancer, the International Society of Nephrology, WHO kidney disease estimates, and national CKD registries, can also provide valuable insights into the burden of CKD attributable to GN in WCBA. However, disparities in global coverage and data collection methods, particularly in low-SDI regions, may lead to variations between estimates derived from different sources. We advocate for future efforts by WHO and International Society of Nephrology to establish specific codes for CKD in WCBA and recommend incorporating renal function checks into prenatal care in low-SDI settings to improve data quality and ultimately reduce the burden of CKD attributable to GN. Another limitation of this study is its reliance on national-level estimates from the GBD. Subnational variations in the burden of chronic kidney disease attributable to glomerulonephritis within countries could not be assessed, as accessing such data typically requires collaboration with national disease control centers (CDC) or specific registries. To address this gap, we plan to conduct a future in-depth study focused specifically on China to investigate internal regional disparities in the burden of chronic kidney disease attributable to glomerulonephritis among WCBA. Moreover, the projections from the Bayesian model are based on extrapolating past trends (1990–2021) and assume constant relationships between risk factors and disease burden. Potential future shifts in public health policies, healthcare advancements, or the emergence of new risk factors (e.g., pandemics) could affect the accuracy of these predictions.^[61]^ This limits the ability to analyze CKD risk factors and provide recommendations for policy and healthcare. Future studies should incorporate more diverse data and external validation to improve predictions.

## 5. Conclusions

Between 1990 and 2021, both the ASIR and ASDR of CKD attributable to GN in WCBA demonstrated an upward trajectory. The disease burden was notably higher across both younger and older age groups. From the perspective of growth trends, the burden of CKD attributable to GN among WCBA across nearly all age groups since 2018 has exhibited a notable acceleration in growth rates, potentially attributable to the impact of the COVID-19 pandemic. Importantly, regions with higher SDI exhibited a substantially lower disease burden compared to those with lower SDI. Frontier analysis identified the countries most urgently in need of interventions to address CKD attributable to GN in WCBA. According to the BAPC model, the global disease burden of CKD in WCBA attributable to GN is projected to continue rising through 2049. In conclusion, this study offers novel insights into the evolving disease burden of CKD attributable to GN in WCBA, while raising awareness of the vulnerabilities of this specific population. Moreover, it provides robust epidemiological data to support CKD research. The findings have significant implications for policymaking, enabling government agencies to develop targeted interventions for CKD attributable to GN in WCBA, and emphasizing the importance of addressing the needs of vulnerable populations.

## Author contributions

**Conceptualization:** Run-Ze Wang, Ye-Xin Chen, Hong-Fang Liu.

**Data curation:** Run-Ze Wang, Ji-Yuan Hu.

**Formal analysis:** Ye-Xin Chen.

**Funding acquisition:** Yi-Shan Wu.

**Methodology:** Zhe-Yu Xu.

**Project administration:** Bei-Bei Ye.

**Resources:** Mao-Xuan Lin.

**Software:** Dong-Sen Hu.

**Supervision:** Zhao-Xi Dong.

**Visualization:** Jia-You Liu.

**Writing – original draft:** Hong-Fang Liu.

**Writing – review & editing:** Hong-Fang Liu.






