# Supplementary material for: Global burden and projections of chronic kidney disease attributable to chronic glomerulonephritis in women of childbearing age
Source: Medicine (Baltimore). 2026 Jul 17;105(29):e49583. doi: 10.1097/MD.0000000000049583 (PMC13384626; doi:10.1097/MD.0000000000049583)
Supplement: Supplementary file 1 [file medi-105-e49583-s001.docx]

**Supplementary Table 1** ASR, APC and AAPC of DALYs and Incidence of CKD attributable to GN in WCBA by different age groups from 1990 to 2021 by Joinpoint regression.

| **Ages** | **Joinpoint** | **Segment** | **Segment.Start** | **Segment.End** | **APC_95CI** |
| --- | --- | --- | --- | --- | --- |
| **DALYs** | | | | | |
| 15 to 19 | 4 | 0 | 1990 | 1994 | 1.26(0.97 to 1.73) |
| 15 to 19 | 4 | 1 | 1994 | 2000 | 0.19(-0.06 to 0.39) |
| 15 to 19 | 4 | 2 | 2000 | 2007 | -1.1(-1.32 to -0.96) |
| 15 to 19 | 4 | 3 | 2007 | 2016 | 0.49(0.41 to 0.7) |
| 15 to 19 | 4 | 4 | 2016 | 2021 | -0.06(-0.44 to 0.15) |
| 20 to 24 | 5 | 0 | 1990 | 2000 | 1.32(1.24 to 1.41) |
| 20 to 24 | 5 | 1 | 2000 | 2004 | -0.45(-0.69 to 0.22) |
| 20 to 24 | 5 | 2 | 2004 | 2008 | -1.89(-2.32 to -1.49) |
| 20 to 24 | 5 | 3 | 2008 | 2012 | 0.05(-1.13 to 0.46) |
| 20 to 24 | 5 | 4 | 2012 | 2016 | 1.36(1.02 to 1.79) |
| 20 to 24 | 5 | 5 | 2016 | 2021 | 0.65(0.23 to 0.84) |
| 25 to 29 | 3 | 0 | 1990 | 1997 | -0.01(-0.33 to 0.22) |
| 25 to 29 | 3 | 1 | 1997 | 2002 | 1.97(1.47 to 2.52) |
| 25 to 29 | 3 | 2 | 2002 | 2014 | -0.83(-0.94 to -0.73) |
| 25 to 29 | 3 | 3 | 2014 | 2021 | 1.39(1.17 to 1.7) |
| 30 to 34 | 4 | 0 | 1990 | 1993 | 0.26(-0.66 to 1.41) |
| 30 to 34 | 4 | 1 | 1993 | 1997 | -1.21(-2.11 to 1.2) |
| 30 to 34 | 4 | 2 | 1997 | 2003 | 1.03(0 to 2.12) |
| 30 to 34 | 4 | 3 | 2003 | 2019 | -0.01(-0.82 to 0.12) |
| 30 to 34 | 4 | 4 | 2019 | 2021 | 1.59(0.14 to 2.29) |
| 35 to 39 | 4 | 0 | 1990 | 1995 | 0.71(0.33 to 1.27) |
| 35 to 39 | 4 | 1 | 1995 | 2011 | -0.19(-0.38 to 0.15) |
| 35 to 39 | 4 | 2 | 2011 | 2016 | 1.91(-0.51 to 2.39) |
| 35 to 39 | 4 | 3 | 2016 | 2019 | -0.55(-1 to 2.19) |
| 35 to 39 | 4 | 4 | 2019 | 2021 | 1.14(-0.16 to 1.92) |
| 40 to 44 | 5 | 0 | 1990 | 1997 | 0.23(-0.19 to 0.46) |
| 40 to 44 | 5 | 1 | 1997 | 2002 | 1.33(0.82 to 1.96) |
| 40 to 44 | 5 | 2 | 2002 | 2006 | -1.67(-2.24 to -1.01) |
| 40 to 44 | 5 | 3 | 2006 | 2011 | -0.5(-1.04 to 0.48) |
| 40 to 44 | 5 | 4 | 2011 | 2016 | 2.04(1.7 to 2.75) |
| 40 to 44 | 5 | 5 | 2016 | 2021 | 1.17(0.47 to 1.47) |
| 45 to 49 | 4 | 0 | 1990 | 1996 | -0.16(-0.5 to 0.08) |
| 45 to 49 | 4 | 1 | 1996 | 2005 | 0.93(0.78 to 1.14) |
| 45 to 49 | 4 | 2 | 2005 | 2012 | -0.6(-0.88 to -0.38) |
| 45 to 49 | 4 | 3 | 2012 | 2019 | 0.97(0.48 to 1.15) |
| 45 to 49 | 4 | 4 | 2019 | 2021 | 2.62(1.54 to 3.25) |
| 15 to 19 | 3 | 0 | 1990 | 1998 | -0.86(-1.29 to -0.59) |
| 15 to 19 | 3 | 1 | 1998 | 2001 | 2.7(1.23 to 3.33) |
| 15 to 19 | 3 | 2 | 2001 | 2011 | -1.64(-1.98 to -1.42) |
| 15 to 19 | 3 | 3 | 2011 | 2021 | 0.75(0.52 to 1.02) |
| 20 to 24 | 4 | 0 | 1990 | 1997 | -1.11(-1.41 to -0.81) |
| 20 to 24 | 4 | 1 | 1997 | 2001 | 4.18(3.37 to 5.1) |
| 20 to 24 | 4 | 2 | 2001 | 2011 | -0.31(-0.58 to -0.13) |
| 20 to 24 | 4 | 3 | 2011 | 2015 | 2.46(1.78 to 3.43) |
| 20 to 24 | 4 | 4 | 2015 | 2021 | 0.59(0.01 to 0.89) |
| 25 to 29 | 5 | 0 | 1990 | 1992 | -2.82(-3.59 to -1.43) |
| 25 to 29 | 5 | 1 | 1992 | 1997 | -0.77(-1.03 to 2.42) |
| 25 to 29 | 5 | 2 | 1997 | 2002 | 3.05(2.28 to 3.59) |
| 25 to 29 | 5 | 3 | 2002 | 2010 | 0.47(0.2 to 0.72) |
| 25 to 29 | 5 | 4 | 2010 | 2017 | 2.8(2.57 to 3.09) |
| 25 to 29 | 5 | 5 | 2017 | 2021 | -0.05(-0.46 to 0.42) |
| 30 to 34 | 4 | 0 | 1990 | 1997 | -1.29(-1.6 to -0.98) |
| 30 to 34 | 4 | 1 | 1997 | 2001 | 3.86(3.15 to 4.97) |
| 30 to 34 | 4 | 2 | 2001 | 2008 | 0(-0.5 to 0.32) |
| 30 to 34 | 4 | 3 | 2008 | 2016 | 2.8(2.55 to 3.25) |
| 30 to 34 | 4 | 4 | 2016 | 2021 | 0.55(-0.04 to 1.01) |
| 35 to 39 | 4 | 0 | 1990 | 1997 | -0.19(-0.53 to 0.12) |
| 35 to 39 | 4 | 1 | 1997 | 2000 | 5.62(4.4 to 6.22) |
| 35 to 39 | 4 | 2 | 2000 | 2009 | -0.17(-0.47 to 0.02) |
| 35 to 39 | 4 | 3 | 2009 | 2016 | 2.9(2.58 to 3.5) |
| 35 to 39 | 4 | 4 | 2016 | 2021 | 0.68(0.09 to 1.15) |
| 40 to 44 | 5 | 0 | 1990 | 1993 | -0.25(-1.36 to 0.52) |
| 40 to 44 | 5 | 1 | 1993 | 1997 | 1.59(1.1 to 4.41) |
| 40 to 44 | 5 | 2 | 1997 | 2001 | 4.95(0.13 to 5.58) |
| 40 to 44 | 5 | 3 | 2001 | 2010 | -0.43(-0.61 to -0.23) |
| 40 to 44 | 5 | 4 | 2010 | 2015 | 2.7(2.23 to 3.33) |
| 40 to 44 | 5 | 5 | 2015 | 2021 | 1.32(0.89 to 1.59) |
| 45 to 49 | 5 | 0 | 1990 | 1993 | -1.4(-2.74 to -0.3) |
| 45 to 49 | 5 | 1 | 1993 | 1997 | 0.51(0.05 to 6.45) |
| 45 to 49 | 5 | 2 | 1997 | 2001 | 6.25(0.32 to 6.92) |
| 45 to 49 | 5 | 3 | 2001 | 2010 | 0.24(-0.04 to 0.64) |
| 45 to 49 | 5 | 4 | 2010 | 2015 | 2.49(1.95 to 3.47) |
| 45 to 49 | 5 | 5 | 2015 | 2021 | 0.84(0.38 to 1.16) |
| 15 to 19 | 2 | 0 | 1990 | 2000 | -0.49(-0.77 to -0.19) |
| 15 to 19 | 2 | 1 | 2000 | 2009 | -3.99(-4.46 to -3.66) |
| 15 to 19 | 2 | 2 | 2009 | 2021 | -0.9(-1.12 to -0.66) |
| 20 to 24 | 2 | 0 | 1990 | 2000 | 0.33(-0.05 to 0.73) |
| 20 to 24 | 2 | 1 | 2000 | 2012 | -3.48(-3.86 to -3.2) |
| 20 to 24 | 2 | 2 | 2012 | 2021 | -0.71(-1.14 to -0.14) |
| 25 to 29 | 2 | 0 | 1990 | 2004 | -0.59(-0.76 to -0.4) |
| 25 to 29 | 2 | 1 | 2004 | 2014 | -3.12(-3.6 to -2.81) |
| 25 to 29 | 2 | 2 | 2014 | 2021 | -0.74(-1.3 to 0.06) |
| 30 to 34 | 3 | 0 | 1990 | 1994 | 1.04(0.23 to 2.37) |
| 30 to 34 | 3 | 1 | 1994 | 1997 | -4.56(-5.35 to -2.8) |
| 30 to 34 | 3 | 2 | 1997 | 2010 | -1.31(-1.49 to -0.25) |
| 30 to 34 | 3 | 3 | 2010 | 2021 | -2.1(-2.59 to -1.83) |
| 35 to 39 | 4 | 0 | 1990 | 1994 | 3.1(2.63 to 3.62) |
| 35 to 39 | 4 | 1 | 1994 | 2003 | -3.01(-3.35 to -2.82) |
| 35 to 39 | 4 | 2 | 2003 | 2012 | -2.14(-2.4 to -1.82) |
| 35 to 39 | 4 | 3 | 2012 | 2015 | 1.33(0.22 to 1.86) |
| 35 to 39 | 4 | 4 | 2015 | 2021 | -1.63(-2.17 to -1.32) |
| 40 to 44 | 4 | 0 | 1990 | 1994 | 2.48(1.81 to 3.56) |
| 40 to 44 | 4 | 1 | 1994 | 1997 | -4.33(-5.19 to -2.74) |
| 40 to 44 | 4 | 2 | 1997 | 2002 | -0.25(-0.99 to 1.3) |
| 40 to 44 | 4 | 3 | 2002 | 2011 | -4.16(-4.65 to -3.83) |
| 40 to 44 | 4 | 4 | 2011 | 2021 | 0.24(-0.06 to 0.62) |
| 45 to 49 | 5 | 0 | 1990 | 1992 | -1.44(-3.5 to 2.25) |
| 45 to 49 | 5 | 1 | 1992 | 1995 | 3.98(-3.43 to 5.33) |
| 45 to 49 | 5 | 2 | 1995 | 1998 | -3.9(-4.8 to -0.6) |
| 45 to 49 | 5 | 3 | 1998 | 2005 | -0.95(-4.48 to 0.6) |
| 45 to 49 | 5 | 4 | 2005 | 2013 | -4.13(-4.85 to -0.91) |
| 45 to 49 | 5 | 5 | 2013 | 2021 | 0.01(-0.61 to 0.74) |
| 15 to 19 | 3 | 0 | 1990 | 1995 | 0.76(0.5 to 1.17) |
| 15 to 19 | 3 | 1 | 1995 | 2000 | -0.2(-0.66 to 0.12) |
| 15 to 19 | 3 | 2 | 2000 | 2009 | -1.41(-1.59 to -1.3) |
| 15 to 19 | 3 | 3 | 2009 | 2021 | 0.29(0.21 to 0.37) |
| 20 to 24 | 4 | 0 | 1990 | 1996 | 0.64(0.41 to 1.07) |
| 20 to 24 | 4 | 1 | 1996 | 1999 | 0.08(-1.47 to 0.55) |
| 20 to 24 | 4 | 2 | 1999 | 2010 | -1.21(-1.52 to 0.76) |
| 20 to 24 | 4 | 3 | 2010 | 2016 | 0.74(-0.22 to 1.27) |
| 20 to 24 | 4 | 4 | 2016 | 2021 | 0.11(-0.36 to 0.47) |
| 25 to 29 | 3 | 0 | 1990 | 2000 | -0.16(-0.27 to -0.03) |
| 25 to 29 | 3 | 1 | 2000 | 2009 | -1.68(-1.84 to -1.54) |
| 25 to 29 | 3 | 2 | 2009 | 2016 | 0.78(0.61 to 1.2) |
| 25 to 29 | 3 | 3 | 2016 | 2021 | -0.25(-0.74 to 0.06) |
| 30 to 34 | 4 | 0 | 1990 | 1996 | -0.02(-0.29 to 0.27) |
| 30 to 34 | 4 | 1 | 1996 | 2005 | -1.37(-1.72 to -0.79) |
| 30 to 34 | 4 | 2 | 2005 | 2011 | -0.55(-1.42 to -0.16) |
| 30 to 34 | 4 | 3 | 2011 | 2014 | 1.03(-0.54 to 1.37) |
| 30 to 34 | 4 | 4 | 2014 | 2021 | 0.22(-0.2 to 0.42) |
| 35 to 39 | 4 | 0 | 1990 | 1995 | 0.27(-0.11 to 0.69) |
| 35 to 39 | 4 | 1 | 1995 | 2006 | -1.38(-1.74 to -1.24) |
| 35 to 39 | 4 | 2 | 2006 | 2011 | -0.46(-1.24 to 0.18) |
| 35 to 39 | 4 | 3 | 2011 | 2014 | 1.25(0.34 to 1.67) |
| 35 to 39 | 4 | 4 | 2014 | 2021 | 0.07(-0.33 to 0.27) |
| 40 to 44 | 3 | 0 | 1990 | 1997 | 0.35(0.22 to 0.49) |
| 40 to 44 | 3 | 1 | 1997 | 2002 | -2.07(-2.41 to -1.84) |
| 40 to 44 | 3 | 2 | 2002 | 2009 | -1.07(-1.26 to -0.84) |
| 40 to 44 | 3 | 3 | 2009 | 2021 | 0.41(0.34 to 0.48) |
| 45 to 49 | 3 | 0 | 1990 | 1997 | -0.26(-0.41 to -0.1) |
| 45 to 49 | 3 | 1 | 1997 | 2008 | -1.17(-1.26 to -1.09) |
| 45 to 49 | 3 | 2 | 2008 | 2016 | -0.08(-0.24 to 0.06) |
| 45 to 49 | 3 | 3 | 2016 | 2021 | 0.96(0.73 to 1.28) |
| 15 to 19 | 2 | 0 | 1990 | 1999 | 0.96(0.78 to 1.16) |
| 15 to 19 | 2 | 1 | 1999 | 2008 | -0.63(-1.22 to -0.46) |
| 15 to 19 | 2 | 2 | 2008 | 2021 | -0.19(-0.29 to 0.02) |
| 20 to 24 | 1 | 0 | 1990 | 1999 | 1.44(1.2 to 1.7) |
| 20 to 24 | 1 | 1 | 1999 | 2021 | -0.42(-0.48 to -0.36) |
| 25 to 29 | 2 | 0 | 1990 | 2001 | 0.81(0.65 to 1.02) |
| 25 to 29 | 2 | 1 | 2001 | 2017 | -0.05(-0.14 to 0.28) |
| 25 to 29 | 2 | 2 | 2017 | 2021 | -0.84(-2.01 to -0.2) |
| 30 to 34 | 1 | 0 | 1990 | 1996 | 0.92(0.6 to 1.4) |
| 30 to 34 | 1 | 1 | 1996 | 2021 | 0.12(0.08 to 0.16) |
| 35 to 39 | 3 | 0 | 1990 | 2002 | 0.67(0.49 to 0.91) |
| 35 to 39 | 3 | 1 | 2002 | 2011 | -0.33(-1.14 to -0.09) |
| 35 to 39 | 3 | 2 | 2011 | 2014 | 2.29(1.06 to 2.89) |
| 35 to 39 | 3 | 3 | 2014 | 2021 | -0.13(-0.69 to 0.16) |
| 40 to 44 | 3 | 0 | 1990 | 2003 | 0.55(0.44 to 0.7) |
| 40 to 44 | 3 | 1 | 2003 | 2012 | -0.15(-0.54 to 0.03) |
| 40 to 44 | 3 | 2 | 2012 | 2015 | 3.13(2.08 to 3.61) |
| 40 to 44 | 3 | 3 | 2015 | 2021 | -0.05(-0.44 to 0.21) |
| 45 to 49 | 4 | 0 | 1990 | 1998 | 0.77(0.09 to 0.97) |
| 45 to 49 | 4 | 1 | 1998 | 2003 | 1.42(-0.24 to 1.98) |
| 45 to 49 | 4 | 2 | 2003 | 2007 | -0.91(-1.55 to 0.31) |
| 45 to 49 | 4 | 3 | 2007 | 2016 | 0.95(0.81 to 1.45) |
| 45 to 49 | 4 | 4 | 2016 | 2021 | 0.17(-0.42 to 0.5) |
| 15 to 19 | 4 | 0 | 1990 | 1995 | 0.73(0.41 to 1.17) |
| 15 to 19 | 4 | 1 | 1995 | 2004 | -0.79(-0.92 to -0.62) |
| 15 to 19 | 4 | 2 | 2004 | 2007 | -2.3(-2.67 to -1.75) |
| 15 to 19 | 4 | 3 | 2007 | 2010 | 1.39(0.65 to 1.77) |
| 15 to 19 | 4 | 4 | 2010 | 2021 | -1.42(-1.53 to -1.34) |
| 20 to 24 | 2 | 0 | 1990 | 2003 | 0.7(0.58 to 0.83) |
| 20 to 24 | 2 | 1 | 2003 | 2009 | -2.48(-3 to -2.08) |
| 20 to 24 | 2 | 2 | 2009 | 2021 | -0.07(-0.2 to 0.08) |
| 25 to 29 | 3 | 0 | 1990 | 1996 | -1.58(-2.31 to -1.08) |
| 25 to 29 | 3 | 1 | 1996 | 2003 | 2.47(1.98 to 3.01) |
| 25 to 29 | 3 | 2 | 2003 | 2014 | -2.24(-2.48 to -2.05) |
| 25 to 29 | 3 | 3 | 2014 | 2021 | 1.63(1.25 to 2.09) |
| 30 to 34 | 5 | 0 | 1990 | 1992 | -0.51(-1.91 to 0.66) |
| 30 to 34 | 5 | 1 | 1992 | 1997 | -3.04(-3.54 to -2.65) |
| 30 to 34 | 5 | 2 | 1997 | 2004 | 2.24(1.93 to 2.54) |
| 30 to 34 | 5 | 3 | 2004 | 2007 | -2.15(-2.53 to -1.01) |
| 30 to 34 | 5 | 4 | 2007 | 2019 | -0.92(-1.06 to -0.67) |
| 30 to 34 | 5 | 5 | 2019 | 2021 | 3.14(1.61 to 3.88) |
| 35 to 39 | 5 | 0 | 1990 | 1996 | 0.08(-0.15 to 0.75) |
| 35 to 39 | 5 | 1 | 1996 | 2008 | -0.37(-0.49 to -0.21) |
| 35 to 39 | 5 | 2 | 2008 | 2011 | -1.1(-1.44 to -0.49) |
| 35 to 39 | 5 | 3 | 2011 | 2016 | 1.28(1.03 to 1.81) |
| 35 to 39 | 5 | 4 | 2016 | 2019 | -0.73(-1.16 to -0.14) |
| 35 to 39 | 5 | 5 | 2019 | 2021 | 1.96(1.06 to 2.71) |
| 40 to 44 | 4 | 0 | 1990 | 1995 | -1.26(-2.29 to -0.56) |
| 40 to 44 | 4 | 1 | 1995 | 2002 | 2.26(1.77 to 2.92) |
| 40 to 44 | 4 | 2 | 2002 | 2005 | -3.23(-3.92 to -1.3) |
| 40 to 44 | 4 | 3 | 2005 | 2011 | -0.99(-1.65 to 0.69) |
| 40 to 44 | 4 | 4 | 2011 | 2021 | 1.39(1.12 to 1.78) |
| 45 to 49 | 4 | 0 | 1990 | 1995 | -0.03(-1.79 to 0.8) |
| 45 to 49 | 4 | 1 | 1995 | 2008 | 1.29(1.11 to 1.96) |
| 45 to 49 | 4 | 2 | 2008 | 2011 | -3.62(-4.3 to -1.86) |
| 45 to 49 | 4 | 3 | 2011 | 2019 | 0.3(-0.56 to 0.81) |
| 45 to 49 | 4 | 4 | 2019 | 2021 | 4.5(1.64 to 5.89) |
| **Incidence** | | | | | |
| 15 to 19 | 5 | 0 | 1990 | 1999 | 0.98(0.92 to 1.04) |
| 15 to 19 | 5 | 1 | 1999 | 2005 | -0.25(-0.33 to 0.13) |
| 15 to 19 | 5 | 2 | 2005 | 2012 | 1.44(-0.17 to 1.54) |
| 15 to 19 | 5 | 3 | 2012 | 2015 | 0.91(0.54 to 1.37) |
| 15 to 19 | 5 | 4 | 2015 | 2019 | -0.02(-0.27 to 0.11) |
| 15 to 19 | 5 | 5 | 2019 | 2021 | 1.18(0.79 to 1.45) |
| 20 to 24 | 5 | 0 | 1990 | 1993 | -0.02(-0.56 to 0.32) |
| 20 to 24 | 5 | 1 | 1993 | 2000 | 1.03(0.93 to 1.19) |
| 20 to 24 | 5 | 2 | 2000 | 2006 | -0.08(-0.26 to 0.04) |
| 20 to 24 | 5 | 3 | 2006 | 2016 | 0.98(0.94 to 1.04) |
| 20 to 24 | 5 | 4 | 2016 | 2019 | -0.4(-0.58 to -0.08) |
| 20 to 24 | 5 | 5 | 2019 | 2021 | 1.28(0.9 to 1.6) |
| 25 to 29 | 5 | 0 | 1990 | 1993 | 0.01(-0.18 to 0.13) |
| 25 to 29 | 5 | 1 | 1993 | 2000 | 0.48(0.44 to 0.56) |
| 25 to 29 | 5 | 2 | 2000 | 2005 | 0.31(0.22 to 0.36) |
| 25 to 29 | 5 | 3 | 2005 | 2015 | 0.79(0.77 to 0.81) |
| 25 to 29 | 5 | 4 | 2015 | 2019 | -0.37(-0.42 to -0.33) |
| 25 to 29 | 5 | 5 | 2019 | 2021 | 1.6(1.46 to 1.72) |
| 30 to 34 | 5 | 0 | 1990 | 1993 | 0.74(0.53 to 0.91) |
| 30 to 34 | 5 | 1 | 1993 | 1999 | -0.02(-0.13 to 0.05) |
| 30 to 34 | 5 | 2 | 1999 | 2005 | 0.55(0.45 to 0.65) |
| 30 to 34 | 5 | 3 | 2005 | 2015 | 1.07(1.04 to 1.11) |
| 30 to 34 | 5 | 4 | 2015 | 2019 | -0.51(-0.59 to -0.43) |
| 30 to 34 | 5 | 5 | 2019 | 2021 | 1.64(1.41 to 1.83) |
| 35 to 39 | 5 | 0 | 1990 | 1997 | 0.82(0.77 to 0.87) |
| 35 to 39 | 5 | 1 | 1997 | 2002 | -0.03(-0.1 to 0.05) |
| 35 to 39 | 5 | 2 | 2002 | 2009 | 0.8(0.73 to 0.85) |
| 35 to 39 | 5 | 3 | 2009 | 2015 | 1.27(1.2 to 1.36) |
| 35 to 39 | 5 | 4 | 2015 | 2019 | 0.48(0.37 to 0.59) |
| 35 to 39 | 5 | 5 | 2019 | 2021 | 1.71(1.49 to 1.9) |
| 40 to 44 | 4 | 0 | 1990 | 1996 | 0.3(0.16 to 0.41) |
| 40 to 44 | 4 | 1 | 1996 | 2001 | 1.14(0.98 to 1.34) |
| 40 to 44 | 4 | 2 | 2001 | 2008 | 0.05(-0.03 to 0.15) |
| 40 to 44 | 4 | 3 | 2008 | 2019 | 1.08(0.16 to 1.11) |
| 40 to 44 | 4 | 4 | 2019 | 2021 | 1.53(1.12 to 1.76) |
| 45 to 49 | 3 | 0 | 1990 | 2001 | 0.31(0.27 to 0.34) |
| 45 to 49 | 3 | 1 | 2001 | 2006 | 1.12(1.01 to 1.24) |
| 45 to 49 | 3 | 2 | 2006 | 2011 | 0.12(0.01 to 0.22) |
| 45 to 49 | 3 | 3 | 2011 | 2021 | 0.87(0.84 to 0.91) |
| 15 to 19 | 2 | 0 | 1990 | 2001 | 0.82(0.74 to 0.93) |
| 15 to 19 | 2 | 1 | 2001 | 2009 | -0.1(-0.45 to 0.04) |
| 15 to 19 | 2 | 2 | 2009 | 2021 | 0.39(0.32 to 0.49) |
| 20 to 24 | 5 | 0 | 1990 | 1994 | 0.03(-0.08 to 0.14) |
| 20 to 24 | 5 | 1 | 1994 | 1999 | 1.56(1.48 to 1.63) |
| 20 to 24 | 5 | 2 | 1999 | 2006 | 0.43(0.37 to 0.53) |
| 20 to 24 | 5 | 3 | 2006 | 2013 | 0.18(0 to 0.24) |
| 20 to 24 | 5 | 4 | 2013 | 2019 | 0.57(0.5 to 0.73) |
| 20 to 24 | 5 | 5 | 2019 | 2021 | -0.3(-0.54 to 0.02) |
| 25 to 29 | 5 | 0 | 1990 | 1994 | -0.71(-0.9 to -0.53) |
| 25 to 29 | 5 | 1 | 1994 | 2001 | 1.65(1.55 to 1.79) |
| 25 to 29 | 5 | 2 | 2001 | 2005 | 0.79(0.45 to 1.17) |
| 25 to 29 | 5 | 3 | 2005 | 2010 | -0.18(-0.53 to 0.02) |
| 25 to 29 | 5 | 4 | 2010 | 2019 | 0.52(0.45 to 0.71) |
| 25 to 29 | 5 | 5 | 2019 | 2021 | -0.27(-0.67 to 0.25) |
| 30 to 34 | 5 | 0 | 1990 | 1995 | -0.34(-0.48 to -0.22) |
| 30 to 34 | 5 | 1 | 1995 | 2000 | 1.43(1.27 to 1.64) |
| 30 to 34 | 5 | 2 | 2000 | 2005 | 1.1(0.4 to 1.2) |
| 30 to 34 | 5 | 3 | 2005 | 2010 | -0.64(-0.74 to -0.49) |
| 30 to 34 | 5 | 4 | 2010 | 2019 | 0.87(0.83 to 0.98) |
| 30 to 34 | 5 | 5 | 2019 | 2021 | 0.22(-0.08 to 0.65) |
| 35 to 39 | 4 | 0 | 1990 | 1995 | 0.18(-0.27 to 0.41) |
| 35 to 39 | 4 | 1 | 1995 | 2005 | 0.83(0.74 to 0.96) |
| 35 to 39 | 4 | 2 | 2005 | 2010 | -1.5(-1.64 to 0.09) |
| 35 to 39 | 4 | 3 | 2010 | 2017 | 1.22(-1.52 to 1.32) |
| 35 to 39 | 4 | 4 | 2017 | 2021 | 1.74(1.4 to 2.31) |
| 40 to 44 | 4 | 0 | 1990 | 1995 | 0.07(-0.62 to 0.38) |
| 40 to 44 | 4 | 1 | 1995 | 2000 | 1.25(0.9 to 1.93) |
| 40 to 44 | 4 | 2 | 2000 | 2005 | 0.33(-1.17 to 0.59) |
| 40 to 44 | 4 | 3 | 2005 | 2010 | -2.49(-2.77 to -2.22) |
| 40 to 44 | 4 | 4 | 2010 | 2021 | 1.52(1.42 to 1.61) |
| 45 to 49 | 5 | 0 | 1990 | 1995 | -0.13(-0.31 to 0.06) |
| 45 to 49 | 5 | 1 | 1995 | 2000 | 1.31(1.12 to 1.59) |
| 45 to 49 | 5 | 2 | 2000 | 2005 | 0.39(0.17 to 0.53) |
| 45 to 49 | 5 | 3 | 2005 | 2010 | -2.3(-2.4 to -2.19) |
| 45 to 49 | 5 | 4 | 2010 | 2019 | 1.26(1.21 to 1.34) |
| 45 to 49 | 5 | 5 | 2019 | 2021 | 0.17(-0.11 to 0.64) |
| 15 to 19 | 5 | 0 | 1990 | 1994 | 0.53(0.18 to 0.75) |
| 15 to 19 | 5 | 1 | 1994 | 1999 | 1.44(1.31 to 1.65) |
| 15 to 19 | 5 | 2 | 1999 | 2006 | -0.51(-0.58 to -0.44) |
| 15 to 19 | 5 | 3 | 2006 | 2015 | 1.09(1.03 to 1.15) |
| 15 to 19 | 5 | 4 | 2015 | 2019 | -0.02(-0.29 to 0.2) |
| 15 to 19 | 5 | 5 | 2019 | 2021 | 0.98(0.52 to 1.3) |
| 20 to 24 | 5 | 0 | 1990 | 1994 | -0.36(-0.54 to -0.16) |
| 20 to 24 | 5 | 1 | 1994 | 2000 | 1.54(1.43 to 1.65) |
| 20 to 24 | 5 | 2 | 2000 | 2007 | -0.05(-0.2 to 0.04) |
| 20 to 24 | 5 | 3 | 2007 | 2015 | 0.57(0.48 to 0.68) |
| 20 to 24 | 5 | 4 | 2015 | 2019 | -0.83(-1.12 to -0.62) |
| 20 to 24 | 5 | 5 | 2019 | 2021 | 0.91(0.38 to 1.24) |
| 25 to 29 | 5 | 0 | 1990 | 1994 | -1.32(-1.47 to -1.18) |
| 25 to 29 | 5 | 1 | 1994 | 2000 | 1.17(1.07 to 1.32) |
| 25 to 29 | 5 | 2 | 2000 | 2009 | 0.6(0.53 to 0.77) |
| 25 to 29 | 5 | 3 | 2009 | 2015 | 0.35(0.12 to 0.45) |
| 25 to 29 | 5 | 4 | 2015 | 2019 | -1.21(-1.39 to -1.06) |
| 25 to 29 | 5 | 5 | 2019 | 2021 | 1.23(0.88 to 1.6) |
| 30 to 34 | 5 | 0 | 1990 | 1996 | -0.59(-0.65 to -0.53) |
| 30 to 34 | 5 | 1 | 1996 | 2000 | 1.03(0.94 to 1.17) |
| 30 to 34 | 5 | 2 | 2000 | 2008 | 0.67(0.57 to 0.71) |
| 30 to 34 | 5 | 3 | 2008 | 2015 | 1.02(0.97 to 1.09) |
| 30 to 34 | 5 | 4 | 2015 | 2019 | -0.75(-0.84 to -0.67) |
| 30 to 34 | 5 | 5 | 2019 | 2021 | 1.29(1.06 to 1.48) |
| 35 to 39 | 5 | 0 | 1990 | 1994 | -0.17(-0.49 to 0.05) |
| 35 to 39 | 5 | 1 | 1994 | 1999 | 1.48(1.34 to 1.68) |
| 35 to 39 | 5 | 2 | 1999 | 2004 | -0.6(-0.84 to -0.46) |
| 35 to 39 | 5 | 3 | 2004 | 2010 | 0.82(0.59 to 1) |
| 35 to 39 | 5 | 4 | 2010 | 2016 | 1.62(1.48 to 1.95) |
| 35 to 39 | 5 | 5 | 2016 | 2021 | 0.87(0.65 to 1.03) |
| 40 to 44 | 5 | 0 | 1990 | 1995 | -0.46(-0.66 to -0.26) |
| 40 to 44 | 5 | 1 | 1995 | 2000 | 2.53(2.36 to 2.72) |
| 40 to 44 | 5 | 2 | 2000 | 2003 | -0.02(-0.36 to 0.89) |
| 40 to 44 | 5 | 3 | 2003 | 2007 | -1.03(-1.49 to -0.72) |
| 40 to 44 | 5 | 4 | 2007 | 2012 | 0.71(0.31 to 1.17) |
| 40 to 44 | 5 | 5 | 2012 | 2021 | 1.74(1.64 to 1.88) |
| 45 to 49 | 5 | 0 | 1990 | 1992 | -1.8(-2.26 to -1.07) |
| 45 to 49 | 5 | 1 | 1992 | 1995 | 0.41(0.12 to 1.68) |
| 45 to 49 | 5 | 2 | 1995 | 1998 | 2.04(1.04 to 2.28) |
| 45 to 49 | 5 | 3 | 1998 | 2007 | 1.03(0.6 to 1.11) |
| 45 to 49 | 5 | 4 | 2007 | 2014 | -0.14(-0.28 to 0.02) |
| 45 to 49 | 5 | 5 | 2014 | 2021 | 1.57(1.46 to 1.7) |
| 15 to 19 | 5 | 0 | 1990 | 1994 | 0.82(0.74 to 0.91) |
| 15 to 19 | 5 | 1 | 1994 | 2004 | -0.5(-0.54 to -0.47) |
| 15 to 19 | 5 | 2 | 2004 | 2007 | 0.3(-0.1 to 0.5) |
| 15 to 19 | 5 | 3 | 2007 | 2015 | 0.82(0.78 to 0.88) |
| 15 to 19 | 5 | 4 | 2015 | 2019 | -0.3(-0.39 to -0.22) |
| 15 to 19 | 5 | 5 | 2019 | 2021 | 2.04(1.82 to 2.24) |
| 20 to 24 | 5 | 0 | 1990 | 1996 | 0.4(0.28 to 0.49) |
| 20 to 24 | 5 | 1 | 1996 | 2005 | -0.6(-0.67 to -0.55) |
| 20 to 24 | 5 | 2 | 2005 | 2010 | 0.53(0.29 to 0.66) |
| 20 to 24 | 5 | 3 | 2010 | 2015 | 1.14(1.01 to 1.36) |
| 20 to 24 | 5 | 4 | 2015 | 2019 | -0.3(-0.48 to -0.15) |
| 20 to 24 | 5 | 5 | 2019 | 2021 | 1.67(1.27 to 1.97) |
| 25 to 29 | 5 | 0 | 1990 | 1997 | 0.46(0.39 to 0.53) |
| 25 to 29 | 5 | 1 | 1997 | 2005 | -0.72(-0.8 to -0.66) |
| 25 to 29 | 5 | 2 | 2005 | 2009 | 0.6(0.15 to 0.77) |
| 25 to 29 | 5 | 3 | 2009 | 2015 | 1.17(1.07 to 1.35) |
| 25 to 29 | 5 | 4 | 2015 | 2019 | -0.11(-0.28 to 0.07) |
| 25 to 29 | 5 | 5 | 2019 | 2021 | 1.84(1.44 to 2.14) |
| 30 to 34 | 5 | 0 | 1990 | 1995 | 0.91(0.83 to 0.98) |
| 30 to 34 | 5 | 1 | 1995 | 2000 | -0.63(-0.71 to -0.56) |
| 30 to 34 | 5 | 2 | 2000 | 2006 | 0.16(0.1 to 0.23) |
| 30 to 34 | 5 | 3 | 2006 | 2015 | 1.03(1 to 1.06) |
| 30 to 34 | 5 | 4 | 2015 | 2019 | -0.12(-0.2 to -0.04) |
| 30 to 34 | 5 | 5 | 2019 | 2021 | 2.06(1.83 to 2.26) |
| 35 to 39 | 5 | 0 | 1990 | 1994 | 0.86(0.55 to 1.21) |
| 35 to 39 | 5 | 1 | 1994 | 2000 | -0.47(-0.89 to -0.29) |
| 35 to 39 | 5 | 2 | 2000 | 2004 | 0.59(-0.08 to 1.05) |
| 35 to 39 | 5 | 3 | 2004 | 2012 | 1.25(1.12 to 1.52) |
| 35 to 39 | 5 | 4 | 2012 | 2019 | 0.17(-0.04 to 0.29) |
| 35 to 39 | 5 | 5 | 2019 | 2021 | 1.99(1.3 to 2.38) |
| 40 to 44 | 5 | 0 | 1990 | 1994 | 0.98(0.66 to 1.27) |
| 40 to 44 | 5 | 1 | 1994 | 2001 | -0.77(-0.99 to -0.65) |
| 40 to 44 | 5 | 2 | 2001 | 2005 | 0.59(-0.01 to 1.07) |
| 40 to 44 | 5 | 3 | 2005 | 2014 | 1.49(1.42 to 1.63) |
| 40 to 44 | 5 | 4 | 2014 | 2019 | -0.09(-0.38 to 0.1) |
| 40 to 44 | 5 | 5 | 2019 | 2021 | 1.44(0.77 to 1.84) |
| 45 to 49 | 5 | 0 | 1990 | 1995 | 0.97(0.81 to 1.13) |
| 45 to 49 | 5 | 1 | 1995 | 2000 | -1.26(-1.5 to -1.07) |
| 45 to 49 | 5 | 2 | 2000 | 2005 | -0.19(-0.48 to 0.05) |
| 45 to 49 | 5 | 3 | 2005 | 2015 | 1.51(1.29 to 1.59) |
| 45 to 49 | 5 | 4 | 2015 | 2018 | 0.24(0.03 to 1.49) |
| 45 to 49 | 5 | 5 | 2018 | 2021 | 1.14(0.7 to 1.65) |
| 15 to 19 | 5 | 0 | 1990 | 1998 | 0.46(0.38 to 0.55) |
| 15 to 19 | 5 | 1 | 1998 | 2005 | -0.14(-0.26 to -0.06) |
| 15 to 19 | 5 | 2 | 2005 | 2010 | 1.37(1.25 to 1.56) |
| 15 to 19 | 5 | 3 | 2010 | 2015 | 0.73(0.57 to 0.93) |
| 15 to 19 | 5 | 4 | 2015 | 2019 | 0.06(-0.21 to 0.22) |
| 15 to 19 | 5 | 5 | 2019 | 2021 | 1.48(1.03 to 1.8) |
| 20 to 24 | 5 | 0 | 1990 | 2000 | 0.2(0.15 to 0.26) |
| 20 to 24 | 5 | 1 | 2000 | 2005 | -0.3(-0.47 to -0.19) |
| 20 to 24 | 5 | 2 | 2005 | 2010 | 1.42(1.31 to 1.59) |
| 20 to 24 | 5 | 3 | 2010 | 2015 | 0.82(0.64 to 0.94) |
| 20 to 24 | 5 | 4 | 2015 | 2019 | -0.51(-0.68 to -0.36) |
| 20 to 24 | 5 | 5 | 2019 | 2021 | 1.48(1.11 to 1.79) |
| 25 to 29 | 4 | 0 | 1990 | 1994 | 1.08(0.76 to 1.3) |
| 25 to 29 | 4 | 1 | 1994 | 2004 | -0.63(-0.7 to -0.58) |
| 25 to 29 | 4 | 2 | 2004 | 2015 | 1.21(1.16 to 1.26) |
| 25 to 29 | 4 | 3 | 2015 | 2019 | -0.95(-1.18 to -0.78) |
| 25 to 29 | 4 | 4 | 2019 | 2021 | 1.48(0.86 to 1.92) |
| 30 to 34 | 5 | 0 | 1990 | 1994 | 2.16(1.87 to 2.48) |
| 30 to 34 | 5 | 1 | 1994 | 2000 | -1.3(-1.63 to -1.11) |
| 30 to 34 | 5 | 2 | 2000 | 2005 | 0.09(-0.39 to 0.54) |
| 30 to 34 | 5 | 3 | 2005 | 2015 | 1.23(1.14 to 1.37) |
| 30 to 34 | 5 | 4 | 2015 | 2019 | -1.08(-1.51 to -0.68) |
| 30 to 34 | 5 | 5 | 2019 | 2021 | 1.98(1.12 to 2.59) |
| 35 to 39 | 4 | 0 | 1990 | 1994 | 1.74(1.44 to 2.01) |
| 35 to 39 | 4 | 1 | 1994 | 2001 | -0.61(-0.82 to -0.5) |
| 35 to 39 | 4 | 2 | 2001 | 2015 | 0.81(0.76 to 0.86) |
| 35 to 39 | 4 | 3 | 2015 | 2019 | -0.59(-0.96 to -0.28) |
| 35 to 39 | 4 | 4 | 2019 | 2021 | 2.48(1.77 to 2.96) |
| 40 to 44 | 4 | 0 | 1990 | 1994 | 1.72(1.27 to 2.11) |
| 40 to 44 | 4 | 1 | 1994 | 2001 | -0.57(-0.83 to -0.39) |
| 40 to 44 | 4 | 2 | 2001 | 2014 | 0.86(0.79 to 0.95) |
| 40 to 44 | 4 | 3 | 2014 | 2019 | -0.43(-1 to -0.17) |
| 40 to 44 | 4 | 4 | 2019 | 2021 | 2.01(1.06 to 2.55) |
| 45 to 49 | 5 | 0 | 1990 | 1995 | 1.61(1.3 to 1.9) |
| 45 to 49 | 5 | 1 | 1995 | 2000 | -1.24(-1.73 to -0.88) |
| 45 to 49 | 5 | 2 | 2000 | 2004 | 0.29(-0.67 to 0.86) |
| 45 to 49 | 5 | 3 | 2004 | 2014 | 1.11(0.98 to 1.38) |
| 45 to 49 | 5 | 4 | 2014 | 2019 | -0.55(-1.08 to -0.26) |
| 45 to 49 | 5 | 5 | 2019 | 2021 | 1.24(0.35 to 1.82) |
| 15 to 19 | 4 | 0 | 1990 | 1999 | 1.48(1.4 to 1.57) |
| 15 to 19 | 4 | 1 | 1999 | 2005 | -0.4(-0.52 to 0.79) |
| 15 to 19 | 4 | 2 | 2005 | 2012 | 2(-0.34 to 2.16) |
| 15 to 19 | 4 | 3 | 2012 | 2015 | 1.12(0.52 to 1.89) |
| 15 to 19 | 4 | 4 | 2015 | 2021 | 0.14(-0.07 to 0.29) |
| 20 to 24 | 5 | 0 | 1990 | 1994 | 0.08(-0.48 to 0.4) |
| 20 to 24 | 5 | 1 | 1994 | 2000 | 1.57(1.34 to 1.86) |
| 20 to 24 | 5 | 2 | 2000 | 2009 | 0.07(-0.07 to 0.2) |
| 20 to 24 | 5 | 3 | 2009 | 2016 | 1.38(0.22 to 1.58) |
| 20 to 24 | 5 | 4 | 2016 | 2019 | -0.29(-0.56 to 1.39) |
| 20 to 24 | 5 | 5 | 2019 | 2021 | 1.03(0.22 to 1.46) |
| 25 to 29 | 5 | 0 | 1990 | 1993 | -0.78(-0.89 to -0.65) |
| 25 to 29 | 5 | 1 | 1993 | 2005 | 0.59(0.56 to 0.62) |
| 25 to 29 | 5 | 2 | 2005 | 2008 | 0.93(0.75 to 1.01) |
| 25 to 29 | 5 | 3 | 2008 | 2015 | 0.44(0.37 to 0.5) |
| 25 to 29 | 5 | 4 | 2015 | 2019 | -0.06(-0.22 to 0.05) |
| 25 to 29 | 5 | 5 | 2019 | 2021 | 1.73(1.52 to 1.93) |
| 30 to 34 | 5 | 0 | 1990 | 1992 | 0.59(0.1 to 1) |
| 30 to 34 | 5 | 1 | 1992 | 1998 | -0.5(-0.72 to -0.41) |
| 30 to 34 | 5 | 2 | 1998 | 2004 | 0.58(0.4 to 0.75) |
| 30 to 34 | 5 | 3 | 2004 | 2014 | 1.29(1.24 to 1.35) |
| 30 to 34 | 5 | 4 | 2014 | 2019 | -0.44(-0.57 to -0.35) |
| 30 to 34 | 5 | 5 | 2019 | 2021 | 1.47(1 to 1.78) |
| 35 to 39 | 5 | 0 | 1990 | 1992 | 0.19(-0.15 to 0.65) |
| 35 to 39 | 5 | 1 | 1992 | 1996 | 1.59(1.44 to 1.85) |
| 35 to 39 | 5 | 2 | 1996 | 2002 | -0.5(-0.64 to -0.4) |
| 35 to 39 | 5 | 3 | 2002 | 2006 | 0.92(0.23 to 1.15) |
| 35 to 39 | 5 | 4 | 2006 | 2016 | 1.41(1.36 to 1.54) |
| 35 to 39 | 5 | 5 | 2016 | 2021 | 0.87(0.71 to 1) |
| 40 to 44 | 3 | 0 | 1990 | 1996 | -0.03(-0.31 to 0.17) |
| 40 to 44 | 3 | 1 | 1996 | 2001 | 1.79(1.53 to 2.17) |
| 40 to 44 | 3 | 2 | 2001 | 2007 | -0.22(-0.49 to -0.05) |
| 40 to 44 | 3 | 3 | 2007 | 2021 | 1.38(1.33 to 1.43) |
| 45 to 49 | 3 | 0 | 1990 | 2001 | 0.14(0.06 to 0.21) |
| 45 to 49 | 3 | 1 | 2001 | 2007 | 2.11(1.96 to 2.27) |
| 45 to 49 | 3 | 2 | 2007 | 2011 | -0.35(-0.8 to -0.07) |
| 45 to 49 | 3 | 3 | 2011 | 2021 | 1.02(0.94 to 1.11) |

AAPC = average annual percent change; APC = average annual percent change; CKD = chronic kidney disease; GN = glomerulonephritis; WCBA = Women of Childbearing Age; DALYs = the Disability-Adjusted Life Years.
