# Supplementary material for: Global burden and projections of chronic kidney disease attributable to chronic glomerulonephritis in women of childbearing age
Source: Medicine (Baltimore). 2026 Jul 17;105(29):e49583. doi: 10.1097/MD.0000000000049583 (PMC13384626; doi:10.1097/MD.0000000000049583)
Supplement: Supplementary file 2 [file medi-105-e49583-s002.docx]

**Supplementary Table 2** Projection of CKD attributable to GN in WCBA by age groups from 2022 to 2049

|  | **DALYs cases** | **Incidence cases** |
| --- | --- | --- |
| 1990 | 722585(716432,728738) | 17950(17121,18779) |
| 1991 | 734959(728763,741154) | 18351(17524,19178) |
| 1992 | 756026(749741,762310) | 18766(17932,19600) |
| 1993 | 775570(769202,781937) | 19202(18358,20046) |
| 1994 | 794743(788295,801191) | 19659(18804,20514) |
| 1995 | 808417(801913,814922) | 20141(19275,21008) |
| 1996 | 822259(815697,828821) | 20633(19755,21511) |
| 1997 | 838228(831601,844856) | 21104(20214,21994) |
| 1998 | 862291(855567,869016) | 21590(20689,22492) |
| 1999 | 888739(881910,895568) | 22060(21147,22973) |
| 2000 | 911241(904324,918158) | 22553(21628,23477) |
| 2001 | 927356(920377,934336) | 23006(22071,23941) |
| 2002 | 945164(938117,952211) | 23456(22511,24402) |
| 2003 | 963399(956283,970516) | 23906(22950,24862) |
| 2004 | 972626(965475,979777) | 24360(23394,25326) |
| 2005 | 979582(972406,986758) | 24830(23853,25807) |
| 2006 | 980381(973203,987559) | 25329(24341,26318) |
| 2007 | 985691(978494,992888) | 25864(24863,26865) |
| 2008 | 997963(990721,1005205) | 26432(25418,27445) |
| 2009 | 1014244(1006943,1021545) | 27013(25987,28040) |
| 2010 | 1022525(1015194,1029856) | 27571(26532,28610) |
| 2011 | 1029384(1022029,1036740) | 28109(27058,29160) |
| 2012 | 1043379(1035975,1050784) | 28649(27586,29712) |
| 2013 | 1066855(1059368,1074342) | 29161(28087,30236) |
| 2014 | 1081423(1073885,1088961) | 29630(28545,30715) |
| 2015 | 1101113(1093506,1108719) | 30041(28947,31134) |
| 2016 | 1129185(1121482,1136889) | 30367(29266,31467) |
| 2017 | 1141136(1133392,1148880) | 30622(29515,31728) |
| 2018 | 1141973(1134227,1149720) | 30888(29775,32001) |
| 2019 | 1156747(1148949,1164544) | 31245(30123,32367) |
| 2020 | 1183638(1175752,1191525) | 31836(30698,32974) |
| 2021 | 1211357(1203369,1219344) | 32355(31190,33519) |
| 2022 | 1257770(1218213,1297328) | 33409(31862,34957) |
| 2023 | 1284438(1229567,1339310) | 33960(32147,35773) |
| 2024 | 1311531(1234745,1388317) | 34530(32331,36729) |
| 2025 | 1338742(1234608,1442876) | 35111(32413,37810) |
| 2026 | 1365512(1229141,1501883) | 35694(32391,38997) |
| 2027 | 1391630(1218273,1564987) | 36272(32266,40277) |
| 2028 | 1418238(1203400,1633075) | 36857(32059,41655) |
| 2029 | 1445933(1185147,1706720) | 37456(31779,43133) |
| 2030 | 1474184(1163005,1785362) | 38061(31421,44701) |
| 2031 | 1502175(1136136,1868213) | 38657(30971,46342) |
| 2032 | 1529350(1103853,1954847) | 39239(30425,48053) |
| 2033 | 1555584(1066220,2044947) | 39780(29765,49794) |
| 2034 | 1582122(1024090,2140153) | 40309(29016,51602) |
| 2035 | 1609459(977594,2241324) | 40839(28186,53493) |
| 2036 | 1637684(926394,2348973) | 41380(27276,55483) |
| 2037 | 1666512(869818,2463205) | 41937(26289,57586) |
| 2038 | 1695329(807285,2583373) | 42504(25213,59795) |
| 2039 | 1724066(738590,2709542) | 43066(24039,62092) |
| 2040 | 1753290(663661,2842919) | 43639(22774,64505) |
| 2041 | 1783707(582195,2985220) | 44242(21419,67066) |
| 2042 | 1815866(493559,3138172) | 44894(19975,69812) |
| 2043 | 1849875(397015,3302736) | 45595(18432,72758) |
| 2044 | 1885479(297316,3479226) | 46332(16774,75890) |
| 2045 | 1922927(195466,3669042) | 47106(14989,79223) |
| 2046 | 1962518(99059,3873973) | 47918(13063,82773) |
| 2047 | 2004347(13228,4095699) | 48770(10983,86558) |
| 2048 | 2048580(0,4336086) | 49663(8856,90594) |
| 2049 | 2095677(0,4597767) | 50602(6939,94908) |

CKD = chronic kidney disease; GN = glomerulonephritis; WCBA = Women of Childbearing Age; DALYs = the Disability-Adjusted Life Years.
