# Supplementary material for: Global burden and projections of chronic kidney disease attributable to chronic glomerulonephritis in women of childbearing age
Source: Medicine (Baltimore). 2026 Jul 17;105(29):e49583. doi: 10.1097/MD.0000000000049583 (PMC13384626; doi:10.1097/MD.0000000000049583)
Supplement: Supplementary file 3 [file medi-105-e49583-s003.docx]

**Supplementary Table 3** Cases, ASR and AAPC of CKD attributable to GN in WCBA by countries in 2021.

|  | **Incidence** | | | | | **DALYs** | | | | |
| --- | --- | --- | --- | --- | --- | --- | --- | --- | --- | --- |
| **Countries** | **CASE** | **ASIR** | **95%UI** | **AAPC** | **95%UI** | **CASE** | **ASDR** | **95%UI** | **AAPC** | **95%UI** |
| Afghanistan | 283 | 3.83 | (1.44,8.1) | 1.13 | (1.11,1.14) | 7922 | 118.45 | (49.87,252.33) | -0.03 | (-0.14,0.13) |
| Albania | 13 | 2.09 | (0.74,4.5) | 1.8 | (1.76,1.84) | 175 | 27.59 | (17.15,43.1) | -1.32 | (-1.56,-1.13) |
| Algeria | 321 | 2.81 | (1.07,5.99) | 1.14 | (1.12,1.15) | 5073 | 43.72 | (24.94,73.26) | 0.3 | (0.26,0.34) |
| American Samoa | 1 | 4.52 | (1.62,9.71) | 1.73 | (1.69,1.76) | 9 | 79.94 | (42.62,140.98) | 2.79 | (2.72,2.86) |
| Andorra | 0 | 0.59 | (0.18,1.42) | 0.22 | (0.2,0.24) | 4 | 15.9 | (8.91,26.08) | -0.55 | (-0.67,-0.47) |
| Angola | 78 | 1.01 | (0.32,2.22) | 0.72 | (0.69,0.74) | 11355 | 154.07 | (78.58,267.41) | 0.02 | (-0.11,0.2) |
| Antigua and Barbuda | 1 | 2.97 | (1.09,6.41) | 1.53 | (1.5,1.57) | 49 | 195.11 | (142.78,251.45) | 0.66 | (0.42,0.84) |
| Argentina | 114 | 0.93 | (0.33,1.99) | 0.43 | (0.41,0.44) | 4116 | 33.64 | (22.25,48.22) | -1.34 | (-1.52,-1.14) |
| Armenia | 15 | 2.25 | (0.79,4.97) | 0.79 | (0.77,0.81) | 248 | 31.78 | (19.27,52.54) | 1.87 | (1.56,2.16) |
| Australia | 52 | 0.81 | (0.28,1.83) | 0.73 | (0.7,0.74) | 1267 | 18.92 | (13.42,25.76) | 0.26 | (0.15,0.36) |
| Austria | 14 | 0.67 | (0.21,1.55) | 0.34 | (0.32,0.35) | 344 | 15.78 | (9.85,24.16) | -0.21 | (-0.31,-0.1) |
| Azerbaijan | 76 | 3.05 | (1.09,6.56) | 1.15 | (1.12,1.18) | 1323 | 47.46 | (28.11,75.95) | 0.2 | (0.06,0.35) |
| Bahamas | 3 | 2.87 | (1.11,6.05) | 1.25 | (1.23,1.26) | 313 | 285.4 | (181.92,411.66) | 1.26 | (1.04,1.44) |
| Bahrain | 8 | 2.42 | (0.93,5.19) | 1.2 | (1.18,1.22) | 118 | 35.52 | (20.8,56.72) | 0.31 | (0.2,0.41) |
| Bangladesh | 453 | 1 | (0.36,2.16) | 1.54 | (1.51,1.56) | 14009 | 30.89 | (17.44,52.52) | -1.29 | (-1.38,-1.2) |
| Barbados | 2 | 2.6 | (1,5.52) | 1.49 | (1.48,1.51) | 116 | 156.66 | (105.48,226.97) | 0.99 | (0.82,1.11) |
| Belarus | 38 | 1.79 | (0.66,3.89) | 1.43 | (1.39,1.47) | 520 | 21.15 | (14.39,29.35) | 1.24 | (1.06,1.4) |
| Belgium | 18 | 0.66 | (0.21,1.51) | 0.19 | (0.18,0.21) | 448 | 16.54 | (9.35,27.87) | 0.01 | (-0.11,0.13) |
| Belize | 4 | 3.4 | (1.29,7.07) | 1.74 | (1.7,1.77) | 364 | 311.71 | (216.74,424.79) | 1.79 | (1.49,2.06) |
| Benin | 64 | 1.92 | (0.7,4.17) | 0.65 | (0.64,0.66) | 3793 | 121.58 | (67.94,200.25) | 0.03 | (-0.05,0.08) |
| Bermuda | 0 | 1.97 | (0.72,4.32) | 1.27 | (1.25,1.29) | 10 | 70.87 | (51.17,95.42) | -0.01 | (-0.17,0.15) |
| Bhutan | 5 | 2.31 | (0.88,5.02) | 0.94 | (0.92,0.97) | 123 | 61.34 | (29.44,110.74) | -0.18 | (-0.25,-0.09) |
| Bolivia (Plurinational State of) | 47 | 1.53 | (0.57,3.29) | 0.83 | (0.8,0.86) | 6648 | 216.99 | (125.18,346.98) | -0.48 | (-0.54,-0.43) |
| Bosnia and Herzegovina | 13 | 1.71 | (0.59,3.7) | 1.24 | (1.23,1.26) | 190 | 23.97 | (15.88,35.59) | -1.15 | (-1.32,-0.98) |
| Botswana | 11 | 1.68 | (0.62,3.64) | 1.63 | (1.59,1.67) | 564 | 83.42 | (45.81,149.44) | 0.76 | (0.57,0.97) |
| Brazil | 1037 | 1.69 | (0.98,2.59) | 0.35 | (0.31,0.38) | 51549 | 83.23 | (63.67,101.44) | -1.32 | (-1.51,-1.2) |
| Brunei Darussalam | 2 | 1.43 | (0.5,3.1) | 0.57 | (0.56,0.59) | 53 | 40.76 | (25.07,62.7) | -0.97 | (-1.05,-0.9) |
| Bulgaria | 34 | 2.41 | (0.9,5.25) | 2.12 | (2.1,2.14) | 935 | 57.37 | (35.89,90.36) | 1.28 | (1.04,1.47) |
| Burkina Faso | 86 | 1.57 | (0.58,3.34) | 0.41 | (0.38,0.44) | 9625 | 178.92 | (96.7,290.77) | 0.51 | (0.44,0.58) |
| Burundi | 23 | 0.74 | (0.22,1.68) | -0.35 | (-0.37,-0.34) | 4147 | 135.66 | (79.41,216.75) | -0.83 | (-0.89,-0.76) |
| Cabo Verde | 2 | 1.65 | (0.59,3.54) | 1.16 | (1.13,1.18) | 89 | 60.51 | (36.02,95.24) | -0.58 | (-0.67,-0.47) |
| Cambodia | 77 | 1.73 | (0.64,3.75) | 0.44 | (0.44,0.45) | 2681 | 59.44 | (29.67,109.95) | -0.76 | (-0.81,-0.72) |
| Cameroon | 212 | 2.67 | (1.01,5.73) | 0.34 | (0.33,0.36) | 10522 | 137.33 | (67.52,240.62) | -0.6 | (-0.73,-0.5) |
| Canada | 68 | 0.75 | (0.26,1.73) | 0.14 | (0.09,0.18) | 2726 | 30.52 | (22.3,39.87) | 1.98 | (1.89,2.09) |
| Central African Republic | 18 | 1.24 | (0.43,2.71) | 0.9 | (0.87,0.92) | 2555 | 195.71 | (104.25,347.4) | 0.39 | (0.25,0.54) |
| Chad | 64 | 1.62 | (0.59,3.49) | 0.31 | (0.29,0.33) | 3424 | 95.32 | (51.65,167.36) | 0.28 | (0.19,0.39) |
| Chile | 48 | 0.98 | (0.34,2.14) | 0.85 | (0.82,0.87) | 1143 | 23.17 | (15.46,32.44) | -0.98 | (-1.16,-0.8) |
| China | 3436 | 1.03 | (0.54,1.64) | -0.19 | (-0.22,-0.16) | 47094 | 14.2 | (8.76,21) | -2.13 | (-2.19,-2.09) |
| Colombia | 181 | 1.38 | (0.5,3.04) | 0.07 | (0.03,0.1) | 7601 | 57.49 | (40.61,77.94) | -2.44 | (-2.67,-2.25) |
| Comoros | 2 | 1.08 | (0.35,2.44) | 0.4 | (0.37,0.44) | 390 | 201.53 | (95.84,326.88) | -0.18 | (-0.69,0.31) |
| Congo | 24 | 1.63 | (0.56,3.57) | 0.86 | (0.84,0.88) | 3894 | 279.97 | (148.63,479.14) | 0.66 | (0.49,0.87) |
| Cook Islands | 0 | 3.39 | (1.34,7.13) | 1.68 | (1.66,1.69) | 1 | 21.03 | (11.55,35.34) | 0.04 | (0,0.07) |
| Costa Rica | 30 | 2.31 | (0.88,4.85) | 0.78 | (0.77,0.78) | 1342 | 101.09 | (70.31,136) | 0.86 | (0.64,1.05) |
| Coted'Ivoire | 154 | 2.28 | (0.84,4.88) | 0.55 | (0.54,0.56) | 3686 | 57.21 | (29.73,100.31) | 0.43 | (0.36,0.5) |
| Croatia | 14 | 1.47 | (0.52,3.11) | 1.37 | (1.35,1.39) | 265 | 26.73 | (17.33,41.34) | -0.91 | (-1.12,-0.74) |
| Cuba | 55 | 2.16 | (0.78,4.63) | 1.23 | (1.21,1.25) | 2202 | 82.21 | (57.35,109.09) | 0.27 | (0.08,0.46) |
| Cyprus | 3 | 0.67 | (0.21,1.55) | 0.15 | (0.14,0.16) | 61 | 14.59 | (8.55,23.46) | -0.99 | (-1.1,-0.86) |
| Czechia | 33 | 1.36 | (0.47,2.98) | 1.35 | (1.34,1.36) | 479 | 18.58 | (12.37,27.27) | -1.25 | (-1.36,-1.12) |
| Democratic People's Republic of Korea | 112 | 1.7 | (0.59,3.7) | 0.74 | (0.74,0.75) | 1779 | 26.6 | (15.01,44.28) | -0.24 | (-0.25,-0.22) |
| Democratic Republic of the Congo | 218 | 1.03 | (0.34,2.23) | 0.75 | (0.73,0.77) | 34780 | 171.91 | (95.65,282.41) | 0.03 | (-0.08,0.14) |
| Denmark | 9 | 0.67 | (0.21,1.51) | 0.21 | (0.19,0.22) | 177 | 13.1 | (7.27,22.63) | 0.01 | (-0.07,0.07) |
| Djibouti | 3 | 0.86 | (0.28,2.01) | 0.66 | (0.64,0.67) | 398 | 124.97 | (63.78,217.33) | 0.97 | (0.76,1.18) |
| Dominica | 1 | 3.4 | (1.26,7.09) | 0.94 | (0.91,0.97) | 45 | 276.25 | (171.41,414.09) | 1.98 | (1.91,2.04) |
| Dominican Republic | 70 | 2.42 | (0.94,5.16) | 2.08 | (2.05,2.11) | 5790 | 200.78 | (127.57,293.29) | 1.22 | (1.09,1.34) |
| Ecuador | 103 | 2.21 | (0.77,4.8) | 1.45 | (1.43,1.46) | 6243 | 134.02 | (80.58,222.75) | -1.22 | (-1.49,-0.97) |
| Egypt | 745 | 2.94 | (1.1,6.11) | 1.19 | (1.16,1.21) | 15607 | 61.47 | (33.95,104.37) | 0.46 | (0.34,0.6) |
| El Salvador | 47 | 2.67 | (1.02,5.72) | 1.7 | (1.66,1.75) | 4930 | 279.38 | (167.54,428.35) | 2.37 | (2.1,2.63) |
| Equatorial Guinea | 5 | 1.28 | (0.42,2.9) | 1.65 | (1.64,1.67) | 786 | 225.65 | (96.34,429.85) | 1 | (0.84,1.18) |
| Eritrea | 16 | 0.95 | (0.31,2.16) | 0.59 | (0.57,0.61) | 2245 | 143.1 | (73.81,256.78) | 0.2 | (0.13,0.27) |
| Estonia | 6 | 2.02 | (0.75,4.26) | 1.48 | (1.44,1.52) | 214 | 73.11 | (50.79,105.48) | -0.31 | (-0.84,0.18) |
| Eswatini | 6 | 1.96 | (0.7,4.24) | 0.41 | (0.36,0.46) | 489 | 169.92 | (78.15,314.44) | 1.22 | (1.12,1.32) |
| Ethiopia | 201 | 0.75 | (0.34,1.34) | -0.64 | (-0.68,-0.62) | 31337 | 112.41 | (69.48,172.75) | -2.36 | (-2.4,-2.32) |
| Fiji | 7 | 3.08 | (1.18,6.51) | 0.73 | (0.69,0.75) | 104 | 45.6 | (24.27,77.81) | 1 | (0.92,1.08) |
| Finland | 8 | 0.68 | (0.22,1.49) | 1.31 | (1.27,1.34) | 128 | 10.4 | (5.26,18.75) | 0.18 | (0.12,0.24) |
| France | 106 | 0.69 | (0.21,1.64) | 0.54 | (0.52,0.56) | 1488 | 9.62 | (6.41,14.01) | -0.26 | (-0.32,-0.21) |
| Gabon | 7 | 1.53 | (0.52,3.36) | 1.41 | (1.39,1.44) | 1204 | 258.64 | (114.25,460.02) | 1.22 | (1.09,1.39) |
| Gambia | 12 | 1.97 | (0.73,4.2) | 0.73 | (0.71,0.75) | 724 | 125.19 | (64.28,208.96) | 0.64 | (0.4,0.94) |
| Georgia | 20 | 2.69 | (0.98,5.88) | 1.1 | (1.08,1.13) | 407 | 49.11 | (29.24,78.91) | 1.54 | (1.29,1.8) |
| Germany | 120 | 0.66 | (0.2,1.55) | -0.02 | (-0.03,-0.01) | 3028 | 16.13 | (10.33,24.32) | -0.34 | (-0.43,-0.25) |
| Ghana | 202 | 2.18 | (0.78,4.62) | 0.67 | (0.65,0.68) | 16693 | 188.75 | (110.04,297.92) | 0.98 | (0.92,1.02) |
| Greece | 19 | 0.78 | (0.26,1.79) | 0.19 | (0.18,0.2) | 502 | 19.74 | (13.17,29.4) | 0.12 | (0.03,0.2) |
| Greenland | 0 | 0.65 | (0.21,1.42) | 0.19 | (0.17,0.2) | 4 | 30.09 | (19.4,43.38) | 0.23 | (0.18,0.28) |
| Grenada | 1 | 3.49 | (1.3,7.63) | 1.65 | (1.63,1.67) | 85 | 332.98 | (227.76,460.34) | 0.36 | (0.1,0.54) |
| Guam | 1 | 3.66 | (1.38,7.95) | 1.45 | (1.4,1.5) | 14 | 37.96 | (19.09,65.61) | 1.25 | (1.1,1.42) |
| Guatemala | 104 | 2.46 | (0.92,5.34) | 1.46 | (1.44,1.48) | 11537 | 277.41 | (188.56,379.27) | 1.16 | (0.88,1.45) |
| Guinea | 69 | 2.02 | (0.71,4.42) | 0.53 | (0.52,0.54) | 4915 | 154.68 | (87.45,250.63) | -0.16 | (-0.27,-0.05) |
| Guinea-Bissau | 13 | 2.45 | (0.87,5.26) | 0.41 | (0.4,0.42) | 925 | 186.56 | (100.43,318.23) | -0.67 | (-0.71,-0.63) |
| Guyana | 6 | 3.21 | (1.21,6.84) | 1.6 | (1.58,1.62) | 796 | 397.65 | (237.96,602.65) | 2.41 | (2.14,2.69) |
| Haiti | 91 | 2.58 | (0.96,5.63) | 0.62 | (0.6,0.65) | 9237 | 263.27 | (119.41,667.99) | -0.06 | (-0.24,0.05) |
| Honduras | 71 | 2.59 | (0.99,5.46) | 1.04 | (1.03,1.05) | 2920 | 111.81 | (58.91,191.65) | 0.22 | (0.07,0.35) |
| Hungary | 33 | 1.46 | (0.51,3.15) | 1.7 | (1.68,1.71) | 382 | 15.91 | (10.21,23.65) | -1.54 | (-1.69,-1.4) |
| Iceland | 0 | 0.56 | (0.17,1.31) | -0.19 | (-0.2,-0.17) | 9 | 10.48 | (6.39,17.28) | 0.73 | (0.67,0.78) |
| India | 6005 | 1.59 | (0.91,2.42) | -0.3 | (-0.36,-0.25) | 176630 | 47.82 | (32.21,68.32) | -0.14 | (-0.24,-0.06) |
| Indonesia | 1379 | 1.84 | (1.01,2.83) | 0.71 | (0.7,0.72) | 41024 | 54.96 | (31.8,88.01) | -0.34 | (-0.36,-0.32) |
| Iran (Islamic Republic of) | 738 | 2.97 | (1.74,4.45) | 0.76 | (0.72,0.81) | 4169 | 17.51 | (11.23,25.7) | -0.17 | (-0.26,-0.08) |
| Iraq | 345 | 3.34 | (1.27,7.11) | 0.9 | (0.88,0.92) | 4542 | 44.89 | (23.23,81.48) | -1.15 | (-1.2,-1.11) |
| Ireland | 10 | 0.74 | (0.25,1.61) | -0.76 | (-0.79,-0.72) | 184 | 14.21 | (7.86,23.98) | 0.11 | (0.02,0.21) |
| Israel | 18 | 0.8 | (0.26,1.77) | 0.38 | (0.37,0.4) | 388 | 16.98 | (10.21,27) | -0.28 | (-0.37,-0.2) |
| Italy | 72 | 0.52 | (0.23,0.94) | -0.25 | (-0.26,-0.23) | 2058 | 15.14 | (10.12,21.54) | -0.53 | (-0.6,-0.46) |
| Jamaica | 25 | 3.27 | (1.25,7.01) | 1.63 | (1.61,1.66) | 1787 | 231 | (142.78,350.21) | 2 | (1.8,2.17) |
| Japan | 246 | 0.89 | (0.43,1.51) | -0.28 | (-0.34,-0.23) | 1063 | 3.72 | (2.26,5.66) | -0.52 | (-0.55,-0.49) |
| Jordan | 83 | 2.73 | (1.04,5.75) | 0.84 | (0.82,0.86) | 1471 | 48.62 | (28.73,76.51) | -1.28 | (-1.44,-1.08) |
| Kazakhstan | 130 | 2.91 | (1.05,6.34) | 0.68 | (0.66,0.71) | 1941 | 39.27 | (24.27,60.03) | -0.9 | (-1.07,-0.71) |
| Kenya | 116 | 0.9 | (0.46,1.43) | 0.93 | (0.91,0.96) | 17348 | 140.97 | (88.62,212.07) | 1.01 | (0.96,1.05) |
| Kiribati | 2 | 5.01 | (1.78,11.35) | 0.99 | (0.97,1.01) | 17 | 53 | (26.59,96.96) | 0.69 | (0.67,0.71) |
| Kuwait | 39 | 2.57 | (0.99,5.32) | 0.25 | (0.23,0.27) | 304 | 19.61 | (12.48,29.2) | -2.59 | (-2.85,-2.27) |
| Kyrgyzstan | 60 | 3.53 | (1.23,7.46) | 0.21 | (0.19,0.23) | 902 | 52.26 | (33.48,77.03) | -0.26 | (-0.55,0.13) |
| Lao People's Democratic Republic | 46 | 2.34 | (0.86,4.96) | 0.38 | (0.37,0.39) | 1518 | 75.81 | (37.95,137.15) | -0.56 | (-0.6,-0.52) |
| Latvia | 7 | 1.92 | (0.68,4.09) | 1.66 | (1.63,1.69) | 208 | 46.77 | (29.94,71.21) | 0.78 | (0.48,1.16) |
| Lebanon | 39 | 2.63 | (1,5.42) | 1.03 | (1.02,1.05) | 434 | 28.38 | (16.4,45.79) | -1.5 | (-1.61,-1.39) |
| Lesotho | 10 | 1.88 | (0.69,4.05) | 1.52 | (1.49,1.55) | 776 | 170.85 | (83.12,303.37) | 3.79 | (3.63,3.94) |
| Liberia | 30 | 2.11 | (0.79,4.39) | 0.75 | (0.74,0.75) | 2639 | 192.8 | (99.54,324.48) | 0.44 | (0.35,0.52) |
| Libya | 66 | 3.25 | (1.31,6.92) | 1.41 | (1.37,1.44) | 1370 | 66.41 | (32.5,117.99) | 1.28 | (1.15,1.36) |
| Lithuania | 10 | 1.72 | (0.63,3.83) | 1.02 | (0.99,1.04) | 292 | 45.16 | (29.31,67.95) | 0.5 | (0.06,0.83) |
| Luxembourg | 1 | 0.73 | (0.24,1.67) | 0.46 | (0.44,0.48) | 28 | 16.25 | (9.46,26.78) | -0.32 | (-0.39,-0.22) |
| Madagascar | 65 | 0.89 | (0.28,2) | 0.47 | (0.44,0.49) | 10288 | 145.73 | (83.86,236.36) | -0.08 | (-0.13,-0.02) |
| Malawi | 36 | 0.73 | (0.23,1.69) | 0.2 | (0.17,0.23) | 8922 | 186.75 | (102.28,300.75) | 0.05 | (-0.05,0.14) |
| Malaysia | 155 | 1.88 | (0.69,4.08) | 1.1 | (1.09,1.11) | 2785 | 33.26 | (19.11,52.34) | -0.16 | (-0.3,-0.04) |
| Maldives | 2 | 1.83 | (0.66,3.93) | 0.27 | (0.25,0.28) | 36 | 31.31 | (18.05,50.24) | -2.71 | (-2.8,-2.63) |
| Mali | 99 | 1.8 | (0.64,3.76) | 0.21 | (0.19,0.23) | 7578 | 147.32 | (83.83,238.93) | -0.54 | (-0.6,-0.5) |
| Malta | 1 | 0.77 | (0.25,1.7) | 0.64 | (0.63,0.65) | 21 | 20 | (12.12,31.24) | 0.62 | (0.51,0.78) |
| Marshall Islands | 1 | 4.3 | (1.65,9.14) | 1.12 | (1.1,1.14) | 10 | 69.54 | (22.79,199.6) | 1.9 | (1.86,1.92) |
| Mauritania | 25 | 2.33 | (0.87,5.05) | 0.37 | (0.35,0.39) | 1299 | 128.74 | (62.45,234.82) | -0.63 | (-0.71,-0.56) |
| Mauritius | 13 | 3.93 | (1.56,8.01) | 1.55 | (1.53,1.56) | 310 | 97.25 | (56.02,151.17) | 1.43 | (0.98,1.85) |
| Mexico | 1229 | 3.47 | (2.25,4.99) | 1.54 | (1.5,1.58) | 94858 | 264.49 | (171.65,392.2) | 1.19 | (1.05,1.32) |
| Micronesia (Federated States of) | 1 | 5.15 | (1.85,10.77) | 1.32 | (1.3,1.35) | 18 | 70.83 | (36.4,128.42) | 1.47 | (1.43,1.51) |
| Monaco | 0 | 0.66 | (0.2,1.52) | 0.32 | (0.31,0.32) | 1 | 16.14 | (9.25,26.27) | 0.73 | (0.7,0.75) |
| Mongolia | 29 | 3.7 | (1.39,7.97) | 0.49 | (0.47,0.51) | 518 | 59.79 | (35.34,93.84) | -0.9 | (-1.02,-0.77) |
| Montenegro | 3 | 2.16 | (0.77,4.72) | 1.51 | (1.49,1.53) | 69 | 45.26 | (27.9,68.04) | -0.18 | (-0.37,-0.03) |
| Morocco | 285 | 2.93 | (1.14,6.21) | 1.78 | (1.77,1.8) | 2977 | 30.27 | (16.39,52.95) | 0.69 | (0.64,0.74) |
| Mozambique | 54 | 0.74 | (0.23,1.72) | 0.75 | (0.73,0.76) | 9424 | 130.01 | (70.59,214.27) | 0.75 | (0.71,0.79) |
| Myanmar | 403 | 2.65 | (0.97,5.61) | 0.46 | (0.44,0.48) | 10847 | 71.4 | (37.66,120.07) | -1.49 | (-1.54,-1.43) |
| Namibia | 8 | 1.21 | (0.42,2.67) | 0.2 | (0.18,0.22) | 384 | 62.4 | (34.16,113.43) | 0.14 | (0.08,0.2) |
| Nauru | 0 | 4.26 | (1.56,9.33) | 0.93 | (0.9,0.97) | 2 | 70.08 | (35.56,125.06) | 1.25 | (1.21,1.29) |
| Nepal | 259 | 2.88 | (1.08,6.18) | 2.13 | (2.1,2.15) | 5317 | 62.05 | (35.25,101.48) | 0.28 | (0.23,0.34) |
| Netherlands | 22 | 0.55 | (0.17,1.27) | 0.53 | (0.51,0.54) | 526 | 13.42 | (8.49,20.95) | -0.15 | (-0.22,-0.08) |
| New Zealand | 14 | 1.11 | (0.45,2.26) | 0.77 | (0.75,0.78) | 366 | 28.79 | (20.39,38.1) | 0.76 | (0.43,1.14) |
| Nicaragua | 45 | 2.53 | (0.99,5.3) | 1.32 | (1.3,1.35) | 3403 | 192.04 | (122.05,279.26) | 0.52 | (0.28,0.73) |
| Niger | 86 | 1.57 | (0.56,3.34) | 0.08 | (0.06,0.1) | 3966 | 79.81 | (39.93,137.55) | -0.89 | (-0.96,-0.83) |
| Nigeria | 931 | 1.65 | (0.92,2.52) | -0.08 | (-0.1,-0.06) | 50205 | 94.83 | (55.5,158.52) | -0.97 | (-1.02,-0.92) |
| Niue | 0 | 3.53 | (1.34,7.57) | 1.22 | (1.2,1.25) | 0 | 80.34 | (34.18,169.35) | 3.03 | (2.7,3.5) |
| North Macedonia | 11 | 1.94 | (0.68,4.21) | 1.8 | (1.79,1.81) | 187 | 32.64 | (20.01,52.72) | -0.59 | (-0.7,-0.5) |
| Northern Mariana Islands | 1 | 5.22 | (1.97,10.92) | 0.81 | (0.78,0.85) | 5 | 42.64 | (21.52,74.79) | 0.26 | (0.2,0.32) |
| Norway | 7 | 0.51 | (0.21,0.95) | 0.41 | (0.38,0.43) | 144 | 11.01 | (7.29,15.99) | 0.26 | (0.2,0.32) |
| Oman | 21 | 2.08 | (0.82,4.32) | 1.42 | (1.38,1.46) | 331 | 32.26 | (18.66,53.02) | 0.53 | (0.4,0.68) |
| Pakistan | 1626 | 2.66 | (1.36,4.53) | 1.11 | (1.1,1.12) | 61616 | 106.1 | (59.48,175.82) | 1.5 | (1.43,1.56) |
| Palau | 0 | 4.8 | (1.84,10.16) | 1.35 | (1.32,1.37) | 3 | 76.94 | (32.91,145.19) | 2.28 | (2.24,2.31) |
| Palestine | 32 | 2.59 | (1,5.49) | 0.58 | (0.56,0.6) | 442 | 35.54 | (20.97,56.82) | -0.73 | (-0.78,-0.66) |
| Panama | 21 | 1.96 | (0.73,4.06) | 0.86 | (0.84,0.87) | 1307 | 122.12 | (82.27,168.82) | 1.25 | (1,1.46) |
| Papua New Guinea | 46 | 1.74 | (0.63,3.67) | 0.63 | (0.62,0.65) | 667 | 25.43 | (13.37,44.1) | 0.62 | (0.55,0.69) |
| Paraguay | 38 | 2.06 | (0.8,4.32) | 1.24 | (1.23,1.25) | 1987 | 107.19 | (67.2,160.18) | 0.25 | (0.03,0.43) |
| Peru | 156 | 1.64 | (0.62,3.41) | 1.47 | (1.45,1.48) | 13358 | 138.52 | (86.32,208.36) | -0.41 | (-1.08,0) |
| Philippines | 856 | 2.91 | (1.82,4.17) | 1.83 | (1.8,1.86) | 21757 | 74.11 | (44.76,115.49) | 0.78 | (0.72,0.84) |
| Poland | 99 | 1.05 | (0.49,1.77) | -0.19 | (-0.2,-0.17) | 1774 | 18.08 | (12.66,25.62) | -3.28 | (-3.43,-3.16) |
| Portugal | 18 | 0.66 | (0.2,1.54) | 0.28 | (0.26,0.3) | 506 | 18.97 | (12.4,27.73) | -0.51 | (-0.62,-0.36) |
| Puerto Rico | 18 | 2.36 | (0.9,4.97) | 1.36 | (1.33,1.39) | 1029 | 132.3 | (100.99,169.59) | -0.01 | (-0.29,0.22) |
| Qatar | 14 | 2.51 | (0.93,5.56) | 0.83 | (0.8,0.86) | 134 | 23.41 | (14.12,36.86) | -0.88 | (-1.32,-0.41) |
| Republic of Korea | 92 | 0.71 | (0.24,1.54) | -0.97 | (-1.01,-0.93) | 1719 | 13.23 | (8.75,19.02) | -4.39 | (-4.57,-4.24) |
| Republic of Moldova | 15 | 1.7 | (0.62,3.74) | 2.09 | (2.04,2.15) | 339 | 34.39 | (23.97,47.69) | 0.68 | (0.43,1.03) |
| Romania | 65 | 1.54 | (0.54,3.3) | 1.02 | (1,1.03) | 1153 | 25.21 | (17.64,36.18) | -3.06 | (-3.47,-2.77) |
| Russian Federation | 927 | 2.59 | (1.49,3.98) | 1.02 | (1,1.05) | 13259 | 34.82 | (25.85,44.63) | -2.8 | (-2.95,-2.64) |
| Rwanda | 25 | 0.73 | (0.22,1.67) | -0.55 | (-0.58,-0.53) | 5158 | 151.86 | (62.41,253.59) | -1.93 | (-1.99,-1.87) |
| Saint Kitts and Nevis | 0 | 2.73 | (1.05,5.6) | 0.67 | (0.65,0.68) | 31 | 189.72 | (123.25,265.63) | -1.53 | (-1.71,-1.33) |
| Saint Lucia | 1 | 2.96 | (1.1,6.33) | 1.38 | (1.36,1.4) | 115 | 238.72 | (163.31,327.16) | 0.48 | (0.36,0.6) |
| Saint Vincent and the Grenadines | 1 | 3.06 | (1.19,6.53) | 1.36 | (1.34,1.39) | 93 | 326.93 | (223.05,456.07) | 1.69 | (1.36,1.93) |
| Samoa | 2 | 4.05 | (1.51,8.78) | 1.15 | (1.11,1.18) | 26 | 53.59 | (27.66,94.26) | 1.34 | (1.31,1.37) |
| San Marino | 0 | 0.6 | (0.18,1.41) | 0.26 | (0.25,0.27) | 1 | 11.47 | (6.19,19.49) | 0.06 | (-0.04,0.12) |
| Sao Tome and Principe | 2 | 3.76 | (1.35,8.08) | 1.19 | (1.18,1.2) | 145 | 274.58 | (143.46,483.8) | 0.36 | (-0.01,0.75) |
| Saudi Arabia | 482 | 4.77 | (1.83,10.06) | 2.06 | (2,2.12) | 12068 | 113.28 | (58.83,195.19) | 1.66 | (1.63,1.69) |
| Senegal | 72 | 1.83 | (0.66,3.85) | 0.16 | (0.13,0.18) | 4917 | 131.48 | (70.82,223.17) | -0.78 | (-0.95,-0.61) |
| Serbia | 34 | 1.6 | (0.57,3.56) | 1.56 | (1.55,1.59) | 542 | 24.43 | (15.68,35.93) | -1.59 | (-1.78,-1.37) |
| Seychelles | 1 | 2.47 | (0.89,5.37) | 1.17 | (1.16,1.18) | 15 | 61.82 | (34.26,102.67) | 1.29 | (1.07,1.53) |
| Sierra Leone | 46 | 1.98 | (0.71,4.3) | 0.75 | (0.74,0.77) | 2404 | 110.37 | (60.04,183.72) | 0.68 | (0.61,0.75) |
| Singapore | 18 | 1.1 | (0.39,2.41) | 0.09 | (0.07,0.1) | 398 | 25.23 | (16.56,36.13) | -1.78 | (-1.99,-1.59) |
| Slovakia | 19 | 1.4 | (0.49,3.03) | 1.05 | (1.03,1.06) | 294 | 21.17 | (13.21,32.36) | -1.03 | (-1.23,-0.86) |
| Slovenia | 6 | 1.36 | (0.47,3) | 1.55 | (1.53,1.56) | 81 | 16.61 | (10.94,25.18) | -1.72 | (-1.96,-1.47) |
| Solomon Islands | 10 | 5.28 | (1.89,11.46) | -0.49 | (-0.51,-0.48) | 80 | 47.33 | (25.31,82.25) | 0.61 | (0.51,0.71) |
| Somalia | 48 | 0.95 | (0.31,2.13) | 0.21 | (0.18,0.25) | 8799 | 192.86 | (102.55,339.84) | -0.04 | (-0.06,-0.01) |
| South Africa | 276 | 1.78 | (0.95,2.85) | -0.04 | (-0.07,-0.02) | 20007 | 126.06 | (81.27,180.29) | -0.69 | (-1.08,-0.26) |
| South Sudan | 18 | 0.78 | (0.25,1.75) | 0.56 | (0.55,0.58) | 5398 | 237.8 | (123.24,409) | 1.72 | (1.56,1.91) |
| Spain | 56 | 0.48 | (0.15,1.1) | -0.89 | (-0.93,-0.86) | 1579 | 13.93 | (8.1,22.7) | -0.86 | (-0.96,-0.73) |
| Sri Lanka | 105 | 1.84 | (0.66,4.04) | 1.03 | (1.01,1.05) | 1909 | 33.82 | (18.73,56.36) | -0.76 | (-0.95,-0.59) |
| Sudan | 242 | 2.24 | (0.86,4.61) | 0.97 | (0.94,1) | 6730 | 62.45 | (30.36,115.5) | 0.33 | (0.3,0.36) |
| Suriname | 4 | 2.93 | (1.11,6.25) | 1.37 | (1.35,1.4) | 484 | 330.13 | (206.94,491.67) | 2.05 | (1.78,2.4) |
| Sweden | 10 | 0.42 | (0.15,0.9) | -0.54 | (-0.56,-0.51) | 262 | 10.93 | (6.96,16.42) | 0.49 | (0.41,0.57) |
| Switzerland | 15 | 0.72 | (0.22,1.63) | -0.32 | (-0.34,-0.3) | 321 | 14.7 | (7.22,26.26) | -0.26 | (-0.31,-0.21) |
| Syrian Arab Republic | 113 | 2.71 | (1.04,5.74) | 0.56 | (0.54,0.58) | 2205 | 56.97 | (29.85,100.72) | -0.84 | (-0.96,-0.72) |
| Taiwan (Province of China) | 100 | 1.64 | (0.64,3.42) | 1.05 | (1.03,1.06) | 931 | 15.86 | (9.67,24.42) | -1.44 | (-1.51,-1.35) |
| Tajikistan | 58 | 2.26 | (0.78,4.93) | 0.78 | (0.75,0.81) | 670 | 26.6 | (15.23,43.78) | -0.07 | (-0.16,0.02) |
| Thailand | 353 | 2.12 | (0.81,4.53) | 0.59 | (0.57,0.6) | 6851 | 41.48 | (23.45,68.28) | -0.24 | (-0.48,-0.01) |
| Timor-Leste | 6 | 1.77 | (0.65,3.88) | 0.58 | (0.57,0.59) | 176 | 48.83 | (25.83,86.07) | -0.03 | (-0.24,0.16) |
| Togo | 40 | 1.84 | (0.66,3.85) | 0.44 | (0.42,0.45) | 2507 | 119.18 | (63.15,200.23) | -0.26 | (-0.31,-0.21) |
| Tokelau | 0 | 3.87 | (1.37,8.44) | 1.48 | (1.45,1.5) | 0 | 71.07 | (36.38,129.17) | 2.44 | (2.14,2.89) |
| Tonga | 1 | 3.22 | (1.2,6.85) | 0.69 | (0.66,0.71) | 9 | 36.31 | (20.04,61.1) | 0.77 | (0.73,0.8) |
| Trinidad and Tobago | 10 | 2.83 | (1.06,5.88) | 1.54 | (1.5,1.58) | 866 | 241.46 | (151.68,353.61) | 1.88 | (1.65,2.1) |
| Tunisia | 77 | 2.42 | (0.92,5.24) | 1.26 | (1.23,1.29) | 1204 | 36.91 | (21.23,60.97) | 0.28 | (0.22,0.34) |
| Turkey | 472 | 2.11 | (0.81,4.61) | 1.29 | (1.25,1.32) | 5325 | 23.96 | (14.17,38.01) | -1.75 | (-1.86,-1.63) |
| Turkmenistan | 50 | 3.95 | (1.47,8.44) | 1.22 | (1.2,1.25) | 1347 | 107.09 | (62.95,168.59) | 1.11 | (0.91,1.34) |
| Tuvalu | 0 | 3.42 | (1.31,7.13) | 1.11 | (1.08,1.14) | 1 | 49.04 | (25.64,90.33) | 0.67 | (0.64,0.7) |
| Uganda | 51 | 0.53 | (0.17,1.17) | 0.18 | (0.15,0.2) | 13278 | 131.77 | (74.05,213.9) | 1.54 | (1.44,1.64) |
| Ukraine | 169 | 1.71 | (0.64,3.64) | 1.67 | (1.65,1.69) | 4297 | 38.46 | (21.42,60.27) | 5.34 | (4.83,5.88) |
| United Arab Emirates | 63 | 3.24 | (1.23,6.86) | 1.31 | (1.28,1.34) | 557 | 32.39 | (18.02,51.99) | 0.5 | (0.29,0.65) |
| United Kingdom | 118 | 0.74 | (0.34,1.26) | 0.65 | (0.63,0.67) | 2921 | 17.57 | (12.01,24.68) | 0.33 | (0.22,0.42) |
| United Republic of Tanzania | 104 | 0.74 | (0.24,1.65) | 0.18 | (0.15,0.21) | 24700 | 176.15 | (101.7,274.56) | 0.09 | (0.01,0.14) |
| United States Virgin Islands | 0 | 1.13 | (0.61,1.78) | -0.22 | (-0.27,-0.17) | 22 | 60.62 | (49.71,73.09) | 2.4 | (2.34,2.48) |
| United States of America | 905 | 2.66 | (0.98,5.72) | 1.38 | (1.36,1.4) | 48863 | 121.73 | (67.48,194.41) | 0.33 | (0.04,0.67) |
| Uruguay | 7 | 0.79 | (0.25,1.74) | 0.69 | (0.68,0.7) | 202 | 23.23 | (15.24,33.41) | 0.07 | (-0.12,0.23) |
| Uzbekistan | 346 | 4.17 | (1.52,9.29) | 0.61 | (0.58,0.63) | 6595 | 72.73 | (45.73,108.34) | 1.79 | (1.58,2.05) |
| Vanuatu | 3 | 3.39 | (1.3,7.34) | 0.83 | (0.8,0.85) | 33 | 43.27 | (22.55,75.58) | 1.51 | (1.45,1.59) |
| Venezuela (Bolivarian Republic of) | 156 | 2.2 | (0.83,4.55) | 1.16 | (1.13,1.19) | 13389 | 186.03 | (118.32,275.83) | 1.92 | (1.68,2.15) |
| Viet Nam | 447 | 1.78 | (0.65,3.84) | 0.89 | (0.87,0.91) | 10188 | 40.38 | (20.69,67.94) | -0.68 | (-0.71,-0.64) |
| Yemen | 150 | 1.89 | (0.71,3.95) | 0.47 | (0.45,0.5) | 1835 | 24.09 | (12.63,45.63) | -0.09 | (-0.21,0.02) |
| Zambia | 51 | 1.05 | (0.34,2.33) | 0.08 | (0.07,0.1) | 10405 | 221.03 | (118.74,377.83) | -0.65 | (-0.68,-0.61) |
| Zimbabwe | 66 | 1.63 | (0.6,3.46) | 0.92 | (0.88,0.96) | 6479 | 173.3 | (93.91,308.48) | 3.03 | (2.87,3.2) |

ASR = age-standardized rate; AAPC = average annual percentage change; CKD = chronic kidney disease; GN = glomerulonephritis; WCBA = Women of Childbearing Age; SDI = Socio-demographic Index; DALYs = the Disability-Adjusted Life Years; ASIR = age-standardized incidence rate; ASDR = age-standardized DALYs rate.
